# *In vitro* co-culture model of *Trichomonas vaginalis*, *Candida albicans*, and *Lactobacillus crispatus*: a system for assessing antimicrobial activity and microorganism interactions in vaginitis

**DOI:** 10.3389/fpara.2025.1523113

**Published:** 2025-04-14

**Authors:** Fernanda Gomes Cardoso, Luisa Trindade dos Santos, Saulo Almeida Menezes, Graziela Vargas Rigo, Tiana Tasca

**Affiliations:** Faculdade de Farmácia and Centro de Biotecnologia, Universidade Federal do Rio Grande do Sul, Porto Alegre, RS, Brazil

**Keywords:** Candida albicans, co-culture, Lactobacillus crispatus, Trichomonas vaginalis, vaginal microbiota, vaginitis

## Abstract

*Trichomonas vaginalis* is a flagellated protozoan causing trichomoniasis, the most common non-viral sexually transmitted infection. It is associated with various complications, particularly in asymptomatic carriers. Another major cause of vaginitis is *Candida albicans*, a normal member of the vaginal microbiota, which causes vulvovaginal candidiasis when immune imbalances occur, leading to recurrent infections. Treatment-resistant strains of these pathogens pose a significant challenge. *Lactobacillus crispatus*, a dominant species in the vaginal microbiota, produces antimicrobial compounds that help protect the vaginal mucosa. This study establishes an *in vitro* co-culture of *T. vaginalis*, *C. albicans*, and *L. crispatus* to simulate the vaginal microenvironment at the site of infection. MRS medium was chosen for the co-culture, with initial cell densities determined as follows: *T. vaginalis* at 1.0 × 10^6^ trophozoites/mL (counted using a hemocytometer), 3.33 × 10^4^ CFU/mL for *C. albicans*, and either 5.53 × 10^6^ CFU/mL (for co-culture with the ATCC isolate) or 5.53 × 10^7^ CFU/mL (for co-culture with a fresh clinical isolate) for *L. crispatus*. The cell densities of *C. albicans* and *L. crispatus* were quantified as colony-forming units (CFU) on selective agar. The incubation period for co-culture, ensuring optimal growth of all microorganisms, was 24 hours. In co-culture, *L. crispatus* at both tested densities acidified the medium. The co-culture system demonstrated lower MIC values for metronidazole (50 µM in the ATCC isolate co-culture and 25 µM with the fresh clinical isolate) and lower MFC values for fluconazole (6.25 µM), compared to monocultures of *T. vaginalis* (100 µM) and *C. albicans* (12.50 µM). Furthermore, the triple co-culture increased the cytotoxicity to vaginal cell and erythrocytes for the ATCC isolate while significantly inhibited both biofilm formation and metabolic activity of *C. albicans* (by up to 92% and 90%, respectively), as well as its yeast-to-hyphae transition (by up to 70%). SEM analyses highlighted the morphological differences among *T. vaginalis*, *C. albicans*, and *L. crispatus*, including isolate-specific size variations in the protozoan. These findings suggest that this *in vitro* co-culture system is a valuable tool for evaluating the antimicrobial efficacy of novel compounds against vaginitis pathogens and for studying interactions within the vaginal microenvironment.

## 1 Introduction

Low levels of *Lactobacillus* and a diverse microbiota in the vaginal site, indicate dysbiosis, an imbalance between the host and microbiota. Facultative anaerobic bacteria such as *Prevotella bivia*, *Atopobium vaginae*, and *Gardnerella vaginalis* are associated with bacterial vaginosis (BV), the most common dysbiosis condition (Ravel et al., 2011; Gajer et al., 2012). BV elevates the risk of opportunistic infections and sexually transmitted infections (STIs). *Trichomonas vaginalis*, a flagellated extracellular protozoan transmitted sexually, causes the most common non-viral STI globally, known as trichomoniasis. This infection is closely related to this dysbiotic microenvironment (Brotman et al., 2019). According to the World Health Organization (WHO), an estimated 156 million new cases of the infection were reported worldwide (Rowley et al., 2019). Approximately 80% of cases are asymptomatic, promoting chronic infection for years, which can result in complications such as infertility, prostate cancer, cervical cancer, pelvic inflammatory disease, and pregnancy complications (Benchimol et al., 2008; Stark et al., 2009; Edwards et al., 2016; Workowski et al., 2021). Furthermore, *T. vaginalis* presence increases HIV/AIDS transmission and acquisition (Kissinger and Adamski, 2013). FDA-approved drugs for trichomoniasis, namely metronidazole, tinidazole, and secnidazole, belong to the class of 5-nitroimidazoles. Therefore, these drugs share a common mechanism of action and may entail adverse effects (Workowski et al., 2021; Miller and Nyirjesy, 2011; Muzny and Van Gerwen, 2022). Furthermore, the sensitivity of these drugs has been significantly reduced due to resistance mechanisms, reaching concerning rates of 4.3 to 10% of *in vitro* resistance (Kirkcaldy et al., 2012).

In the vaginal microbiota, a fungal community coexists with *Lactobacillus*. The prevalence of fungi was estimated to be between 20-60% in healthy women, pregnant women, and women with diabetes (Bradford and Ravel, 2017). The most prevalent species within this community is *Candida albicans*, comprising approximately 72-91% of the total (Barousse et al., 2004). *Candida* spp. is a dimorphic yeast, capable of acting as a commensal in its yeast form, meaning it does not adversely affect the host, or as an opportunistic pathogen in its hyphal form, leading to vulvovaginal candidiasis (VVC) (Sobel, 1989). Vaginitis caused by *Candida* spp. is the second most prevalent dysbiosis, characterized by an overgrowth of fungi, which induces the transition from yeast to hyphae (Iliev and Underhill, 2013; Powell and Nyirjesy, 2014). Several species of *Candida* are responsible for VVC, with *C. albicans* being the most associated with vaginitis (Farr et al., 2021). Approximately 138 million women are affected by recurrent vulvovaginal candidiasis (RVVC), characterized by four or more episodes of infection per year (Denning et al., 2018). *Candida* spp. isolates show increased drug resistance, especially against fluconazole, due to its fungistatic activity (Bauters et al., 2022). In addition to treatment-related adverse effects, the ineffectiveness of long term use due to isolate resistance is associated with complications such as pelvic inflammatory disease, menstrual disorders, infertility, ectopic pregnancy, spontaneous abortion, and increased susceptibility to HIV (Pasko et al., 1990; Yano et al., 2019).

The predominance of *Lactobacillus* species in the vaginal microbiota represents four out of seven communities-state types (CST), which distinguish several vaginal bacterial communities. These CSTs were established based on next generation sequency technology: CST-I is dominated by *L. crispatus*, CST-II by *L. gasseri*, CST-III by *L. iners*, CST-IV is divided into three subgroups with a diverse range of bacteria, and CST-V by *L. jensenii* (France et al., 2020). The balance between microorganisms in the vaginal microbiota and vaginal cells is referred to as vaginal eubiosis. Approximately 70% of healthy women exhibit a predominance of *Lactobacillus* spp. However, not all *Lactobacillus* species are beneficial or protective, only *L. crispatus* is consistently associated with vaginal eubiosis. *L. crispatus* plays a pivotal role in safeguarding against the development of opportunistic infections and STIs (Witkin et al., 2013; Chee et al., 2020; Kalia et al., 2020).

*In vitro* systems aim to replicate *in vivo* but often lack precision (Lorian, 1989). The co-culture technique, combining different cell types in the same medium to mimic realistic environments, enables cell-cell and drug-cell interactions. This approach enables the creation of an *in vivo*-like cell culture model, which is especially valuable for drug research (Wu et al., 2010; Temkin et al., 2019). Despite its potential, studies that use microbial co-culture to evaluate the antimicrobial activity and drug-microorganism interactions in the context of trichomoniasis and VVC remain scarce. Developing new molecules for treating trichomoniasis and VVC, several research steps are necessary, including preclinical studies involving *in vitro* and *in vivo* tests, as well as clinical studies. A major challenge in advancing treatments for *T. vaginalis* is the lack of a standardized *in vivo* model. The only known susceptible animal is Macaca nemestrina, which developed a persistent infection after a single *T. vaginalis* inoculation (Patton et al., 2006). Furthermore, the treatment of trichomoniasis relies exclusively on drugs within the same class, raising concerns about resistance. Similarly, antifungal drug development faces challenges due to the similarity between fungal and human molecular targets, limiting viable therapeutic options (Lee et al., 2021). In the case of *C. albicans*, azole antifungals have long been the treatment of choice. However, increasing azole resistance and recurrent VVC cases underscores the urgent need to develop new therapeutic agents (Vandecruys et al., 2023). Standardizing *in vitro* methodologies to better simulate *in vivo* conditions could be a promising strategy for advancing drug discovery efforts against trichomoniasis and VVC. In this study we standardized the co-culture of *T. vaginalis*, *C. albicans*, and *L. crispatus* to replicate the vaginal microenvironment. This would enable a single test to evaluate antimicrobial activity *T. vaginalis* and *C. albicans*, as well as determine if the molecule inhibits the growth of *L. crispatus*, representing the healthy microbiota of women. Additionally, this *in vitro* system enables the investigation of interactions among these microorganisms and their influence on virulence factors, proving a more comprehensive platform for therapeutic research.

## 2 Materials and methods

### 2.1 *T. vaginalis* culture and trophozoite densities

The following assays utilized *T. vaginalis* ATCC 30236 and a fresh clinical isolate, TV-LACM6, obtained from the Laboratório de Análises Clínicas e Toxicológicas, Faculdade de Farmácia UFRGS, Brazil (approved by the UFRGS Research Ethical Committee under authorization number 18923). These isolates were selected based on their distinct characteristics; TV-LACM6 exhibits higher hemolytic activity and cytolytic capacity than ATCC 30236 (Menezes et al., 2017). Trophozoites were cultured in Trypticase Yeast Maltose (TYM) medium (Diamond, 1957), supplemented with 10% (v/v) adult bovine serum (ABS) and penicillin/streptomycin, for 24 h under microaerophilic conditions at 37°C. During logarithmic growth, the *T. vaginalis* suspension was centrifuged and resuspended in Man-Rogosa-Sharpe broth (MRS) - chosen based on the results explained in the following sections - (Sigma-Aldrich) (De Man et al., 1960) supplemented with 10% ABS. Trophozoite densities of 1 x 10^6^ (TV1) and 1 x 10^5^ (TV2) trophozoites/mL were tested and adjusted using a Neubauer hemocytometer with Trypan-blue exclusion dye (0.4%) under an optical microscope (Nikon TE 2000-U Eclipse). MRS medium is composed of peptone, meat extract, yeast extract, and glucose, which provide essential nutrients through enzymatic digestion. Growth stimulation is induced by polyoxyethylenesorbitan monooleate, magnesium sulfate, and manganous sulfate. Additionally, ammonium citrate helps maintain a low pH, promoting the growth of *Lactobacillus* while inhibiting the proliferation of other microorganisms.

### 2.2 Fungal and bacterial cultures and densities

*C. albicans* (ATCC 24433) was cultured on Sabouraud dextrose agar for 24 h at 37°C, while *L. crispatus* (CCT 7595), obtained from the Tropical Culture Collection of André Tosello Foundation (PR, Brazil), was grown on MRS agar under the same conditions. The suspensions of *C. albicans* and *L. crispatus* were adjusted to optical densities (OD) of 0.110 and 0.160, respectively, at 620 nm using a Multiskan™ FC Microplate Photometer (Thermo Fischer Scientific, USA). Each suspension was then serially diluted 1:10 in saline, resulting in five different density ranges. The maximum densities used in the co-culture assay were 3.33 x 10^6^ CFU/mL for *C. albicans* (CA1) and 5.53 x 10^7^ CFU/mL for *L. crispatus* (LC1), with minimum densities of 3.33 x 10^2^ CFU/mL (CA5) and 5.53 x 10^3^ CFU/mL (LC5), respectively.

### 2.3 Culture medium for co-culture assay

The media tested for their capacity to support the growth of all three microorganisms in monoculture over 24 h of incubation included TYM, Sabouraud dextrose broth (SDB), and MRS, all supplemented with only 10% ABS, or MRS supplemented with 10% ABS and 20 µM of fluconazole, and RPMI-1640 (Sigma-Aldrich) medium supplemented with 20% fetal bovine serum (FBS). The evaluation of growth was based on the presence or absence of microorganisms, as observed in microscopy slide cultures using Trypan blue dye exclusion.

### 2.4 Checkerboard assay

To determine the optimal density of *T. vaginalis* ATCC 30236, *C. albicans* ATCC 24433, and *L. crispatus* CCT 7595 for co-culture, a checkerboard pattern was created on 96-well culture plates. In this experimental design, one species was tested at a fixed density while the other species was tested across different density ranges. Specifically, *T. vaginalis* was tested at two different levels (1.00 x 10^6^ and 1.00 x 10^5^ trophozoites/mL, TV1 and TV2), while *C. albicans* (3.33 x 10^6^ to 3.33 x 10^2^ CFU/mL, CA1 to CA5) and *L. crispatus* (5.53 x 10^7^ to 5.53 x 10^3^ CFU/mL, LC1 to LC5) were tested across five different density ranges. This approach allowed for the evaluation of various combinations to determine the optimal co-culture density. Monocultures, dual co-cultures, and triple co-culture of *T. vaginalis* isolates, *C. albicans*, and *L. crispatus* were employed, with all combinations detailed in **Supplementary Table 2**. Each adjusted microbial suspension was added in a volume of 50 μL to each well, resulting in a final volume of 200 μL. The plates were then incubated at 37°C for 24 h. All combinations used are shown in **Supplementary Table S2**.

For standardizing the co-culture of TV-LACM6 (at 1.00 x 10^6^ trophozoites/mL), *C. albicans* was maintained at 3.33 x 10^4^ CFU/mL, while *L. crispatus* varied at the first two densities 5.53 x 10^7^ (LC1, OD 0.160) and 5.53 x 10^6^ CFU/mL (LC2, dilution 1:10).

### 2.5 Methods for determination of cell viability and adjustment of standardized density for co-culture assays

After 24 h of co-culture incubation, the contents of each well were diluted 1:1000 in saline. For quantification of *C. albicans* colony formation units (CFU/mL), the diluted samples were inoculated onto Sabouraud agar plates. To assess *L. crispatus* CFU/mL, the samples were inoculated onto MRS agar supplemented with 20 µM fluconazole, a concentration that inhibits *C. albicans* growth. Both sets of agar plates were incubated at 37°C for 24 h. The viability of *T. vaginalis* was evaluated using a Neubauer Hemocytometer and Trypan-blue exclusion dye (0.4%) under an optical microscope (Nikon TE 2000-U Eclipse).

For subsequent assays, *T. vaginalis* isolates were adjusted to a density of 1.00x10^6^ trophozoites/mL (TV1) and *C. albicans* was adjusted to an initial OD of 0.110 before being diluted 1:100 in saline (CA3). *L. crispatus* was adjusted to an initial OD of 0.160 (LC1) and then diluted 1:10 in saline (LC2). These two suspensions of *L. crispatus* were used in co-cultures of *T. vaginalis* isolates TV-LACM6 and ATCC30236, respectively.

### 2.6 Growth kinetics

After standardizing the optimal density for each microorganism, the growth kinetics were assessed. *T. vaginalis* isolates, *C. albicans*, and *L. crispatus* strains were adjusted as described in Section 2.5. To determine the growth curve, each adjusted suspension was added in a volume of 125 μL to each microtube, resulting in a final volume of 500 μL. The growth curve was evaluated in monocultures and co-cultures of the three microorganisms. OD readings were taken at 2, 4, 6, 8, 24, 48, and 72 h. Microbial viability was assessed at these same time points using a hemocytometer for trophozoite viability and OD readings for *C. albicans* and *L. crispatus*.

### 2.7 pH on co-culture

MRS broth supplemented with 10% ABS was prepared with an initial pH of 6.0. *T. vaginalis* isolates, *C. albicans*, and *L. crispatus* strains were adjusted according to the procedure outlined in Section 2.5. To measure the pH of the medium in co-cultures, pH strips were utilized. Each adjusted microbial suspension was added in a volume of 125 μL to each microtube, resulting in a final volume of 500 μL. pH measurements were taken after 24h of incubation, including monocultures, co-cultures of *T. vaginalis* isolates or *C. albicans* strain with two densities of *L. crispatus*, and triple co-cultures.

### 2.8 Drug susceptibility testing with co-culture system

*T. vaginalis* isolates, *C. albicans*, and *L. crispatus* strains were prepared as described in Section 2.5. The effect of metronidazole was analyzed at concentrations ranging from 100 μM to 0.78 μM on monocultures of *T. vaginalis* isolates ATCC 30236 and TV-LACM6, as well as on the co-culture of these isolates with *L. crispatus* at 5.5x10^6^ (dilution 1:10) and 5.5 x 10^7^ CFU/mL (OD 0.160), respectively, and with *C*. *albicans*. The effect of fluconazole was examined at concentrations from 100 μM to 0.78 μM on the monoculture of *C. albicans* and on the co-culture of *C. albicans* with *T. vaginalis* isolate ATCC 30236 and *L. crispatus* at 5.5 x 10^6^ CFU/mL. The effect of penicillin/streptomycin was analyzed at concentrations ranging from 100 μM to 0.78 μM on the monoculture of *L. crispatus* at 5.5 x 10^7^CFU/mL and on the co-culture of *L. crispatus* with *T. vaginalis* isolate TV-LACM6 and *C*. *albicans*. After 24 h of incubation at 37 °C, microbial viability was assessed using the methods described in Section 2.5.

### 2.9 Co-incubation of microbial co-culture with vaginal cells

To create an *in vitro* environment that closely resembles the *in vivo* conditions, a monolayer of vaginal cells from the HMVII lineage (human vaginal epithelial melanoma) was co-incubated with a co-culture of *T. vaginalis* isolates, *C. albicans*, and *L. crispatus*. HMVII cells at density of 2.0 x10^5^ cells/mL were seeded in 96-well culture plates and incubated at 37°C with 5% CO_2_ until they formed a confluent monolayer. The cells were cultured in RPMI medium supplemented with 20% FBS. Subsequently, *T. vaginalis* isolates, *C. albicans*, and *L. crispatus* strains were prepared as described in Section 2.5, with the modification that the medium utilized was RPMI supplemented with 20% FBS. These adjusted microbial suspensions were then added to each well containing the HMVII monolayer. To study the interactions of microorganisms with vaginal epithelial cells, we examined all monoculture and co-culture conditions of *T. vaginalis*, *C. albicans*, and *L. crispatus* at the standard densities described in section 2.5 (TV1, CA3 and LC1 or LC2).

### 2.10 LDH release assay

After the co-incubation of HMVII cell with a co-culture of *T. vaginalis* isolates, *C. albicans*, and *L. crispatus* as described in Section 2.9, an assay was performed to detect the lactate dehydrogenase (LDH) enzyme released by epithelial vaginal cells. This assay aimed to evaluate the cytolytic capacity of *T. vaginalis* isolates in co-culture compared to monoculture. The Cytotox-one Homogeneous Membrane Integrity Assay (Promega, USA) was used for this purpose. The 96-well culture plates were incubated at 37°C with 5% CO_2_ for 6 h. Subsequently, LDH release was measured according to the manufacturers’ instructions. The results were calculated as described by Rigo et al. (2023).

### 2.11 Hemolytic assay

Blood samples obtained from volunteer donors (approved by the UFRGS Research Ethical Committee under authorization number 6742238) were centrifuged (2500 *x* g, 10 min) to separate the plasma, which was then discarded. The erythrocytes were washed three times with phosphate-buffered saline (PBS) at pH 7.0 and adjusted to a density of 5.0 x 10^7^ erythrocytes/mL. To determine hemolytic capacity, each adjusted microbial suspension (as described in Section 2.5) was added in a volume of 125 μL to each microtube, resulting in a final volume of 500 μL. Hemolytic capacity was measured after 24 h at 37°C following the co-incubation of erythrocytes with *T. vaginalis* isolates (monoculture), a co-culture of *T. vaginalis* isolates with either *C. albicans* or *L. crispatus* (dual co-culture), a co-culture with *C. albicans* and *L. crispatus* (dual co-culture), and *T. vaginalis* isolates with *C. albicans* and *L. crispatus* (triple co-culture). Controls, procedures for measuring hemolysis, and result calculations were performed as described by Menezes et al. (2017).

### 2.12 Biofilm biomass evaluation

The biofilm biomass of *C. albicans* and *L. crispatus* was compared with that of *C. albicans* and *L. crispatus* in co-culture. This evaluation was conducted using the crystal violet assay, with 0.4% crystal violet, where absorbance was measured at 570 nm, according to the protocol by Trentin et al. (2011) Briefly, microorganisms were adjusted as described in Section 2.5. The combinations in the 96-well microtiter plate were as follows: only *C. albicans*, only *L. crispatus*, C*. albicans* with *T. vaginalis* isolates, *C. albicans* with *L. crispatus* at two densities, co-culture of all microorganisms, and a sterility control (MRS medium and saline solution). The values obtained for the sterility control were subtracted from all readings, and the results were expressed as a percentage of biofilm formation, with the biofilm formed by only *C. albicans* and *L. crispatus* representing 100%.

### 2.13 Biofilm metabolic viability assay

To evaluate the metabolic viability of biofilm cells formed by the adjusted microorganisms, as described in Section 2.5, the combinations in the 96-well microtiter plate were prepared as outlined in section 2.13. After 24 h, the contents of each well were discarded, and the wells were washed three times with saline, following the method of de Oliveira Dembogurski et al. (2018). Briefly, MTT solution (3-(4,5-dimethyl-thiazol-2-yl)-2,5-diphenyl tetrazolium bromide) at 0.3 mg/mL was added, and the plates were incubated for 1 h 30 min at 37°C. After incubation, MTT was removed, and the wells were washed before adding DMSO (dimethyl sulfoxide). After 20 min, absorbance was measured at 570 nm. The results were calculated as described in Section 2.13.

### 2.14 Yeast to hyphal transition

The morphological transformation of *C. albicans* from yeast to hyphae in co-culture was evaluated following the method described by Toenjes et al. (2005), with some modifications. Briefly, microorganisms were adjusted as described in 2.5 section. The combinations in the 96-well microtiter plate were as follows, only *C. albicans*, C*. albicans* with *T. vaginalis* isolates, *C. albicans* with *L. crispatus* at two densities, and triple co-cultures. The microplate was incubated for 24h at 37°C. After incubation, all combinations were analyzed by counting the number of yeast cells *versus* hyphae under a microscope using a Neubauer hemocytometer. The results were expressed as the hyphae formation rate relative to the total number of *Candida* cells.

### 2.15 Scanning electron microscopy

Co-cultures of *T. vaginalis* isolates, *C albicans* and *L. crispatus*, as well as monocultures of each microorganism, were prepared according to Section 2.5 section. After 24 h, prior to fixation with 2.5% (v/v) glutaraldehyde, the suspensions were washed three times with 1X PBS over 2 h 30 min. Subsequently, a second set of three washes with PBS was performed before post-fixation with 1% osmium tetroxide for 2 h. The samples were then placed on a circular cover slip coated with 0.1% poli-L-lysine. The cover slips were dehydrated in a graded acetone series (30, 50, 70, 80, 95 and 100° GL). Critical point drying was conducted using CO_2,_ and the samples were sputter-coated with gold particles. The metalized samples were then analyzed using a Zeiss EVO MA10 scanning electron microscope. The resulting images were analyzed using the ImageJ software (v1.54m).

### 2.16 Statistical analysis

The data are presented as mean ± standard deviation (SD). Statistical analyses were conducted using the Student’s t-test to compare two groups (monoculture and co-culture) with GraphPad Prism software version 8.0.2. A p-value of < 0.05 was considered statistically significant. The experiments involving checkerboard assay, growth kinetic assay, pH determination in cultures, and biofilm metabolic evaluation were performed in triplicate with at least three independent cultures (n=3). Drug susceptibility testing with the co-culture system, co-incubation of microbial co-cultures with vaginal cells, hemolytic assay, and yeast-to-hyphal transition assessments were performed in triplicate with at least two independent cultures (n=2).

## 3 Results

### 3.1 MRS as the optimal culture medium for co-culture growth

To standardize the co-culture conditions, multiple parameters were evaluated, including the selection of an optimum culture medium, initial densities of *T. vaginalis*, *C. albicans* and *L. crispatus*, incubation time, and cell viability assessment following incubation. Initially, four different media were tested to determine their ability to support the growth of all three microorganisms over a 24h incubation period. Only the presence or absence of growth was assessed (**Supplementary Table S1**). MRS medium with 10% adult bovine serum and RPMI with 20% fetal bovine serum successfully supported microbial growth under these conditions. MRS medium was chosen for experiments without vaginal cells, while RPMI was used when vaginal cells were co-incubated with the microbial co-culture.

### 3.2 Effect of high density of *L. crispatus* on the growth of ATCC 30236 *T. vaginalis* isolate and *C. albicans* after 24 h

To standardize the initial cell density of each microorganism, the viability of *T. vaginalis*, *L. crispatus*, and *C. albicans* was compared between monoculture and co-culture conditions after 24 h of incubation. *T. vaginalis* viability was assessed using Trypan-blue exclusion assay and expressed as trophozoites/mL, while colony-forming units (CFU/mL) for *C. albicans* and *L. crispatus* were determined by colony quantification on selective agar (**Supplementary Table S2**).

When ATCC 30236 *T. vaginalis* isolate was maintained at a constant density of 1.0 x 10^6^ trophozoites/mL (TV1) and co-cultured with *L. crispatus* at varying initial densities (LC1 - LC5; 5.53 x 10^7^ to5.53 x 10^3^ CFU/mL), trichomonads density decreased after 24 h. At the highest *L. crispatus* density (LC1, 5.53 x 10^7^ CFU/mL), *T. vaginalis* exhibited no growth. However, in co-cultures with LC2 (5.53 x 10^6^ CFU/mL), LC3 (5.53 x 10^5^ CFU/mL), LC4 (5.53 x 10^4^ CFU/mL), and LC5 (5.53 x 10^3^ CFU/mL), *T. vaginalis* density increased. At the two lowest *L. crispatus* densities (LC4 and LC5), *T. vaginalis* growth was comparable to the control (monoculture of *T. vaginalis*), with no significant difference observed.

When TV1 was maintained at a constant density and co-cultured with varying initial densities of *C. albicans* (CA1 - CA5; 3.33 x 10^6^ to 3.33 x 10^2^ CFU/mL), only CA1 resulted in a significant change in trophozoite density after 24 h compared to *T. vaginalis* monoculture. The densities of *C. albicans* in co-cultures CA1, CA2, CA3, and CA4 were not significantly different from the *C. albicans* monoculture.

Similarly, when *C. albicans* was maintained at a constant initial density of 3.33 x 10^4^ CFU/mL (CA3) and co-cultured with *L. crispatus*, *C. albicans* viability after 24 h decreased in the presence of LC1 and LC2.The resulting CFU/mL counts were 3.75 ± 2.47 (x10^5^) CFU/mL and 8.25 ± 3.54 (x10^5^) CFU/mL, respectively, compared with the control (*C. albicans* monoculture) at 4.2 ± 1.41 (x10^6^) CFU/mL.

### 3.3 Establishing optimal initial densities for triple co-culture: achieving microbial balance

In triple co-culture, when TV1 (1.0 x 10^6^ trophozoites/mL) was maintained alongside CA3 (3.33 x 10^4^ CFU/mL) and LC2 (5.53 x 10^6^ CFU/mL), no significant difference in microbial densities was observed after 24 h compared to their respective monocultures. The recorded densities were *T. vaginalis* at 26.30 ± 1.34 (x10^5^) trophozoites/mL, *C. albicans* at 28.00 ± 6.36 (x10^5^) CFU/mL, and *L. crispatus* at 80.00 ± 2.83 (x10^5^) CFU/mL. Chequerboard assays, with LC2 maintained at 5.53 x 10^6^ CFU/mL confirmed no significant difference in the TV1 + CA3 + LC2 condition, yielding densities of 22.15 ± 2.15 (x10^5^) trophozoites/mL, 37.00 ± 3.18 (x10^5^) CFU/mL, and 80.00 ± 0.00 (x10^5^) CFU/mL, respectively. When CA3 (3.33 x 10^4^ CFU/ml) was held constant in the TV1 + CA3 + LC2 condition, *T. vaginalis* (24.17 ± 2.49 (x10^5^) trophozoites/mL) and *L. crispatus* (78.00 ± 2.12 (x10^5^) CFU/mL) densities remained unchanged, while *C. albicans* showed a significant difference to 35.00 ± 1.41 (x10^5^) CFU/mL. The TV1 + CA3 + LC4 condition also showed no significant differences compared to monocultures.

Based on these results, the TV1 + CA3 + LC2 combination was selected for further assays as it best mimicked an *in vivo* environment with higher *L. crispatus* density. **Supplementary Table S2** lists all conditions and densities, while **Table 1** summarizes monoculture and selected co-cultures conditions. Subsequent assays compared triple co-culture with dual co-cultures (TV1 + CA3, TV1 + LC2 or CA3 + LC2).

**Table 1:** Initial densities of ATTC 30236 *Trichomonas vaginalis* isolate (TV), *Candida albicans* (CA) and *Lactobacillus crispatus* (LC) for Checkerboard assays.

Fixed MO	Condition or Combination	Initial density (10^6^) Trophozoites/mL^1^ CFU/mL^2^	After 24 h		
			Trophozoites/mL (10^5^) ± SD	*C. albicans* CFU/mL (10^5^) ± SD	*L. crispatus* CFU/mL (10^5^) ± SD
TV1 LC2 CA3	TV1	1.00^1^	30.37 ± 2.32 26.80 ± 2.52 29.75 ± 3.64	- - -	- - -
TV1 CA3	LC1	55.30^2^	- -	- -	101.00 ± 2.12 97.50 ± 6.36
TV1 CA3 LC2	LC2	5.53^2^	- - -	- - -	87.30 ± 1.06 81.30 ± 1.77 88.80 ± 3.18
TV1 CA3 LC2	CA3	0.03^2^	- - -	35.50 ± 0.70 42.00 ± 1.41 37.30 ± 2.47	- - -
TV1 LC2	TV1 + LC2	1.00^1^ + 5.53^2^	13.05 ± 3.61* 17.73 ± 1.42*	- -	65.30 ± 8.13 57.50 ± 0.71*
TV1 CA3	TV1 + CA3	1.00^1^ + 0.03^2^	31.40 ± 2.97 32.20 ± 0.57	39.00 ± 2.83 41.00 ± 0.71	- -
LC2 CA3	LC2 + CA3	5.53^2^ + 0.033^2^	- -	1.50 ± 0.71* 8.25 ± 3.54*	79.30 ± 4.60 62.00 ± 2.83*
TV1 LC2 CA3	TV1 + CA3 + LC2	1.00^1^ + 0.03^2^ + 5.53^2^	26.30 ± 1.34 22.15 ± 2.15 24.17 ± 2.49	28.00 ± 6.36 37.00 ± 3.18 35.00 ± 1.41*	80.00 ± 2.83 80.00 ± 0.00 78.00 ± 2.12

The mean values of densities after 24h for monocultures of TV1, CA3, and LC2 were compared to the mean values of the co-cultures. Statistical significance was set at p < 0.05. Significant differences are indicated by *. All combinations used are shown in **Supplementary Table S2**. TV1: ATCC30236 *T. vaginalis* isolate at an initial density of 1 x 10^6^ trophozoites/mL; CA3: ATCC24433 *C. albicans* isolate at an initial density of 3.3 x 10^4^ CFU/mL; LC2: CCT7595 *L. crispatus* isolate at an initial density 5.53 x 10^6^ CFU/mL.

^1^Trophozoites/mL; ^2^CFU/mL.

Summary of microorganism combinations (monoculture or co-culture) chosen for subsequent assays with mean trophozoites/mL ± SD of TV, mean CFU/mL ± SD CA and LC after 24 h.

For the co-culture standardization using the TV-LACM6 fresh clinical isolate,*T. vaginalis* was maintained at 1x10^6^ trophozoites/mL, co-cultured with *L. crispatus* at its highest density (LC1; 5.53 x 10^7^ CFU/mL), and *C. albicans* (CA3; 3.33 x 10^4^ CFU/mL). Viability (%) was measured with monoculture controls set at 100% after 24h. *T, vaginalis* viability remained at 100% across all conditions remained at 100%. However, in co-culture with TV-LACM6, *C. albicans* and *L. crispatus* viabilities decreased to 58.55% and 85.89%, respectively. In the *L. crispatus* + *C. albicans* co-culture, *L. crispatus* viability was largely unaffected (97.36%), whereas *C. albicans* viability dropped significantly to 4.66%. In triple co-culture, *L. crispatus* viability significantly decreased to 83.12%, while *C. albicans* viability was 86.53% (**Table 2**).

**Table 2:** The mean viability of TV-LACM6 *Trichomonas vaginalis* isolate, ATCC 2433 *Candida albicans* isolate, and *Lactobacillus crispatus* isolate after 24h of co-culture.

Condition	Mean Viability (%) ± SD		
	*T. vaginalis*	*C. albicans*	*L. crispatus*
TV-LACM6	100.00 ± 0.00	–	–
CA3	–	100.00 ± 0.00	–
LC1	–	–	100.00 ± 0.00
TV-LACM6 + CA3	98.58 ± 2.01	58.55 ± 0.73*	
TV-LACM6 + LC1	97.55 ± 3.47	–	85.89 ± 1.07*
CA3 + LC1	–	4.66 ± 0.73*	97.36 ± 3.74
TV-LACM6 + CA3 + LC1	100.39 ± 0.55	86.53 ± 5.13	83.12 ± 2.14*

The mean values of densities after 24h for monocultures of TV-LACM6, CA3, and LC1 were compared to the values of the co-cultures. Statistical significance was set at p < 0.05. Significant differences are indicated by *. All combinations used are shown in **Supplementary Table S2**. (*) Statistically significant difference (p < 0.05). TV-LACM6: *T. vaginalis* fresh clinical isolate at initial density 1.0 x10^6^ trophozoites/mL. CA3: *C. albicans* ATCC 2433 isolate at initial density 3 x 10^4^ CFU/mL. LC1: *L. crispatus* CCT 7595 isolate at initial density 5.53 x 10^7^ CFU/mL (LC1).

### 3.4 Optimal incubation time of co-culture is based on the growth peak of *T. vaginalis i*solates

**Figure 1:**
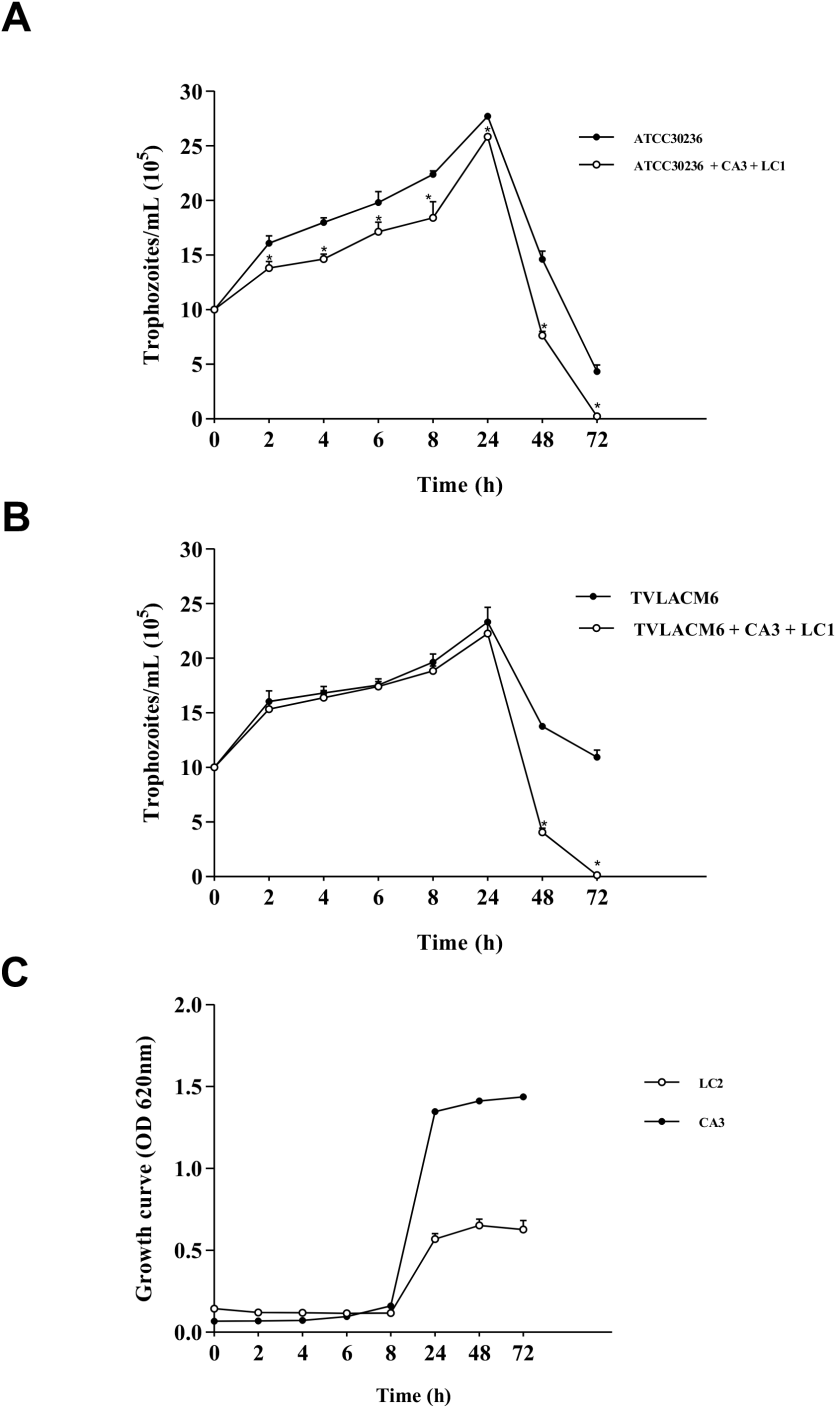
Growth kinetic curves: **(A)** ATCC 30236 *Trichomonas vaginalis* isolate in monoculture or co-culture with *Candida albicans* at 3.3 x 10^4^ CFU/mL (CA3) and *Lactobacillus crispatus* at 5.53 x 10^6^ CFU/mL (LC2). Co-cultured trophozoites were compared with control (monocultured); **(B)** TV-LAM6 *T. vaginalis* isolate in monoculture or co-culture with CA3 and *L. crispatus* at 5.53 x 10^7^ CFU/mL (LC1). Co-cultured trophozoites were compared with control (monocultured). **(C)** *C. albicans* in monoculture (CA3), *L. crispatus* in monoculture (LC2). Data are the mean ± S. D. of at least three different experiments performed in triplicate. *Means statistical difference from control (*P* < 0.05).

The growth kinetics assay revealed that *T. vaginalis* isolates reached their growth peak at 24h in both monoculture and co-culture with *C. albicans* and *L. crispatus*, after which trophozoite density declined (**Figures 1A, B**). In contrast, *C. albicans* and *L. crispatus* peaked at 48 h (**Figure 1C**). Therefore, a 24 h incubation period was chosen for co-culture to ensure all microorganisms were in their active growth phase.

### 3.5 *L. crispatus* acidifies the culture medium

The pH of culture medium was measured after 24 h in all monoculture and co-culture combinations of *T. vaginalis* isolates (ATCC30236 and TV-LACM6, TV1: 1.0 x 10^6^ trophozoites/mL), *C. albicans* (CA3, 3.33 x 10^4^ CFU/mL), and *L. crispatus* (LC1 and LC2, 5.53 x 10^7^ CFU/mL and 5.53 x 10^6^ CFU/mL). The medium alone and the *C. albicans* monoculture had a pH of 6.0. As expected, all combinations involving LC1, as well as the LC2 + CA3, had a pH of 4.0. However, when *T. vaginalis* isolates were present, the pH increased to 5.0 in the TV1 + LC2 and TV1 + CA3 + LC2 co-cultures (**Table 3**). The pH values for co-cultures with ATCC30236 and TV-LACM6 isolates showed no significant differences.

**Table 3:** pH values in monocultures and co-cultures of *Lactobacillus crispatus*, *Candida albicans* and *Trichomonas vaginalis*.

Combination	Monoculture (pH)	Co-culture (pH)	
		*T. vaginalis* ATCC30236	*T. vaginalis* TV-LACM6
LC1	4.0	–	–
LC2	4.0	–	–
CA3	6.0	–	–
TV	–	5.0	5.0
TV + LC1	–	4.0	4.0
TV + LC2	–	5.0	5.0
LC1 + CA3	–	4.0	4.0
LC2 + CA3	–	4.0	4.0
TV + LC1 + CA3	–	4.0	4.0
TV + LC2 + CA3	–	5.0	5.0

LC1, *L. crispatus* at 5.53 x 10^7^ CFU/mL; LC2: *L. crispatus* at 5.53 x 10^6^ CFU/mL; TV, *T. vaginalis* isolates at 1x10^6^ trophozoites/mL; CA3, *C. albicans* remained at 3.3 x 10^4^ CFU/mL (CA3).

### 3.6 Co-culture system as a platform for evaluating drug susceptibility of *T. vaginalis*, C*. albicans*, and *L. crispatus*

As shown in **Table 4**, the minimum inhibitory concentrations (MIC) of *T. vaginalis* isolates were determined using microdilution in microtiter plate wells, while the minimum bactericidal or fungicidal concentrations (MBC or MFC) for *L. crispatus* and *C. albicans* were assessed by sub-culturing on selective agar. Notably, monocultures exhibited higher MIC, MBC or MFC values for metronidazole (MTZ), fluconazole (FLZ), and streptomycin/penicillin (S/P) compared to co-cultures, highlighting the impact of microbial interactions on drug susceptibility.

**Table 4:** Minimum inhibitory concentrations (MIC) of metronidazole for *Trichomonas vaginalis*, minimum fungicidal concentration MFC) of Fluconazole for *Candida albicans*, and minimum bactericidal concentration (MBC) of streptomycin/penicillin for *Lactobacillus crispatus*: Comparative analysis of MIC, MBC, and MFC values and mean trophozoites/mL or CFU/mL after 24 h in monocultures and co-cultures.

Isolate	Metronidazole(µM) MIC^1^		Fluconazole (µM) MFC^2^		Streptomycin/Penicillin (µM) MBC^3^		Mean Trophozoites/mL (10^5^) CFU/mL (10^5^) after 24h		Isolate
	Mono-culture	Co-culture	Mono- culture	Co- culture	Mono- culture	Co-culture	Co-culture	Co-culture Treated^#^	
*T. vaginalis* ATCC30236	100.00^1^	50.00^1^	ND	ND	ND	ND	21.20 ± 0.85^a^ 34.75 ± 1.77^b^ 30.50 ± 4.95^c^	0.00 ± 0.00^a^* 34.75 ± 1.77^b^ 40.25 ± 5.30^c^	TV1 ATCC 30236 ^a^ CA3 ATCC24433 ^b^ LC2 CCT7595 ^c^
*T. vaginalis* TV-LACM6	100.00^1^	25.00^1^	ND	ND	ND	ND	16.00 ± 1.13^d^ 34.75 ± 1.77^b^ 32.50 ± 2.12^e^	0.00 ± 0.00^d^* 34.75 ± 1.77^b^ 68.00 ± 1.41^e^*	TV1 TV-LACM6 ^a^ CA3 ATCC24433 ^b^ LC1 CCT7595 ^c^
*C. albicans* ATCC24433	ND	ND	12.50^2^	6.25^2^	ND	ND	20.00 ± 1.00^a^ 28.75 ± 1.06^b^ 30.00 ± 2.00^c^	20.00 ± 1.00^a^ 0.00 ± 0.00^b^ 31.00 ± 1.00^c^	TV1 ATCC 30236 ^a^ CA3 ATCC24433 ^b^ LC2 CCT7595 ^c^
*L. crispatus* CCT7595	ND	ND	ND	ND	12.5**^3^**	12.5**^3^**	16.00 ± 1.13^d^ 34.75 ± 1.77^b^ 32.50 ± 2.12^e^	19.20 ± 0.71^d^ 36.00 ± 0.77^b^ 00.00 ± 0.00^e^*	TV1 TV-LACM6 ^a^ CA3 ATCC24433 ^b^ LC1 CCT7595 ^c^

^1^MIC, ^2^MFC, ^3^ MBC. ^#^Co-culture treated with MIC for *T. vaginalis* isolates, MFC for *C. albicans*, or MBC for *L. crispatus*.

^a^Trophozoites/mL of *T. vaginalis* ATCC 30236 isolate, initial density at 1 x 10^6^ trophozoites/mL. ^b^CFU/mL of *C. albicans* ATCC 24433 isolate, initial density at 3.33 x 10^4^ CFU/mL. ^c^CFU/mL of *L. crispatus* CCT 7595 isolate (LC2), initial density at 5.53 x 10^6^ CFU/mL. ^d^Trophozoites/mL of *T. vaginalis* TV-LACM6 isolate, initial density at 1 x 10^6^ trophozoites/mL. ^e^ CFU/mL of *L. crispatus* CCT 7595 isolate (LC1), initial density at 5.53 x 10^7^ CFU/mL.

*indicates significant differences.

Interestingly, treatment of *T. vaginalis* isolates with MTZ promoted the growth of *L. crispatus* at both densities (LC1 and LC2), resulting in CFU/mL counts of 40.25 ± 5.30 and 68.00 ± 1.41, respectively-significantly higher that the untreated co-culture values(30.50 ± 4.95 and 32.50 ± 2.12 CFU/mL). Conversely, when *L. crispatus* was treated with S/P, the growth of the TV-LACM6 *T. vaginalis* isolate increased, reaching19.20 ± 0.71 trophozoites/mL, compared to 16.00 ± 1.13 trophozoites/mL in the untreated co-culture. These findings emphasize the importance of the co-culture system in evaluating drug susceptibility, as microbial interactions can significantly influence treatment outcomes.

### 3.7 Co-culture enhances *T. vaginalis* cytolysis and hemolysis

**Figure 2:**
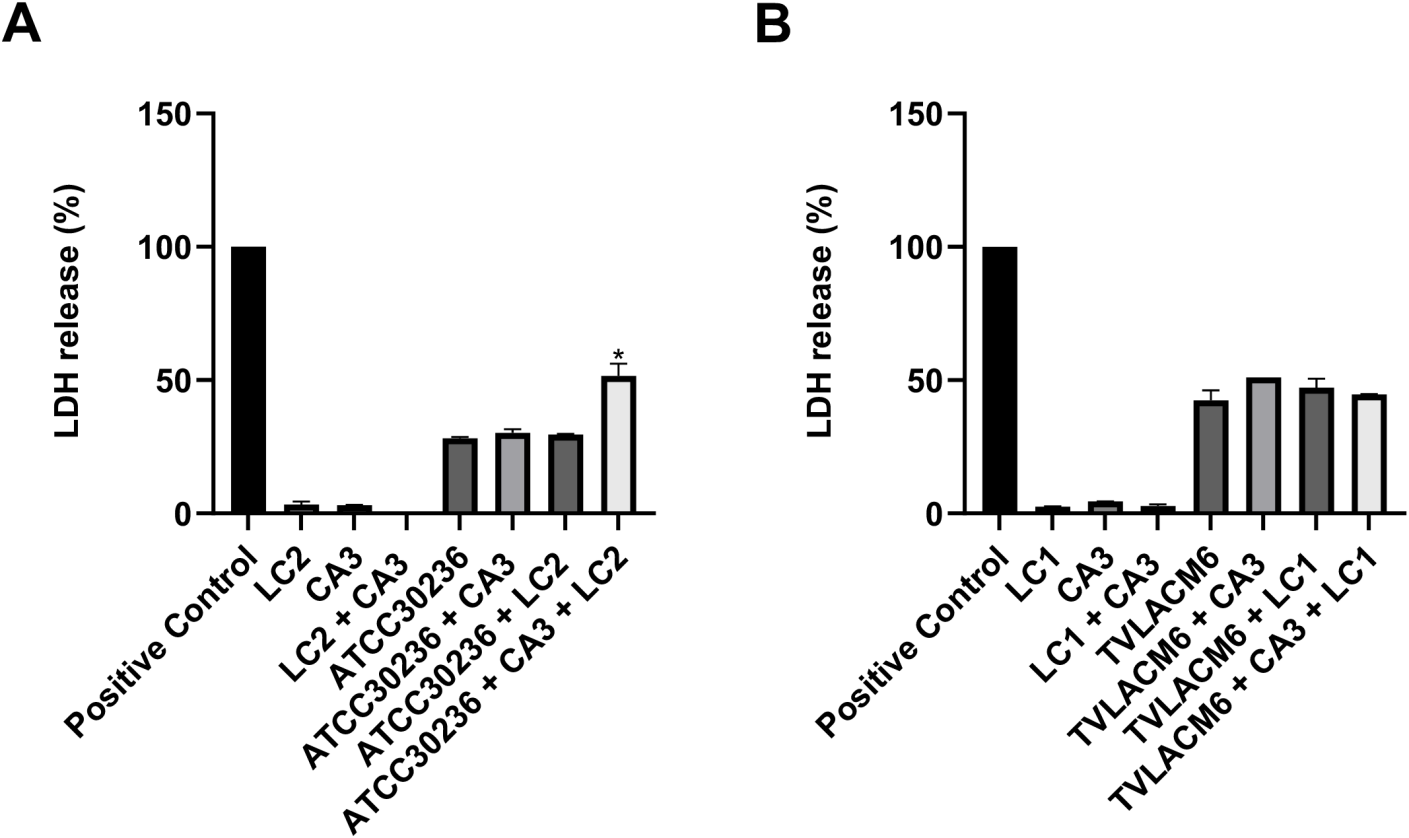
Cytolysis measured by LDH release from human vaginal epithelial cells co-incubated with monocultures or co-cultures of *Trichomonas* vaginalis, *Candida albicans* and *Lactobacillus crispatus.* **(A)** Monocultures of *L. crispatus* (LC2, 5.53 x 10^6^ CFU/mL) *C. albicans* (CA3, 3.33 x 10^4^ CFU/mL), and *T. vaginalis* standard isolate (ATCC30236, 1.0 x 10^6^ trophozoites/mL). Dual co-cultures: LC2 + CA3, ATC30236 + CA3, and ATCC30236 + LC2. Triple co-culture: ATCC30236 + CA3 + LC2. **(B)** Monocultures of *L. crispatus* (LC1, 5.53 x 10^7^ CFU/mL), *C albicans* (CA3), and *T. vaginalis* fresh clinical isolate (TV-LACM6, 1.0 x 10^6^ trophozoites/mL). Dual co-cultures: LC1 + CA3, TV-LACM6 + CA3, and TV-LACM6 + LC1. Triple co-culture: TV-LACM6 + CA3 + LC1. Positive control of cytolysis is HMVII cells treated with 0.2% Triton X-100. Results are expressed as a percentage of total cytolysis. Data are presented as the mean ± S.D. of at least two experiments. The percentage of LDH released from HMVII cells with the monocultures of *T. vaginalis* isolates was compared with that from HMVII cells with co-cultures with the protozoan. (*) indicates a significant difference.

The cytolytic capacity of *T. vaginalis* can be measured by the release of the lactate dehydrogenase (LDH) enzyme from human vaginal epithelial cells (HMVII lineage). **Figure 2A** shows that co-incubation of the co-culture of ATCC*T. vaginalis* isolate (ATCC30236, at initial density 1.0 x 10^6^ trophozoites/mL), *C. albicans* (CA3, 3.33 x 10^4^ CFU/mL), and *L. crispatus* (LC2 5.53 x 10^6^ CFU/mL) with vaginal cells led to a significant increase in the LDH release compared to the co-incubation of ATC30236 alone with vaginal cells. On other hand, it is important to note that the co-incubation of *C. albicans* and two densities of *L. crispatus* (LC1, 5.53 x 10^7^ CFU/mL, and LC2, 5.53 x 10^6^ CFU/mL) showed no interference in LDH release (**Figures 2A, B**). The cytolytic capacity of TV-LACM6 fresh clinical isolate was more pronounced compared with ATCC30236. However, co-culture did not alter this effect, unlike what was observed with the standard isolate (ATCC).

**Figure 3:**
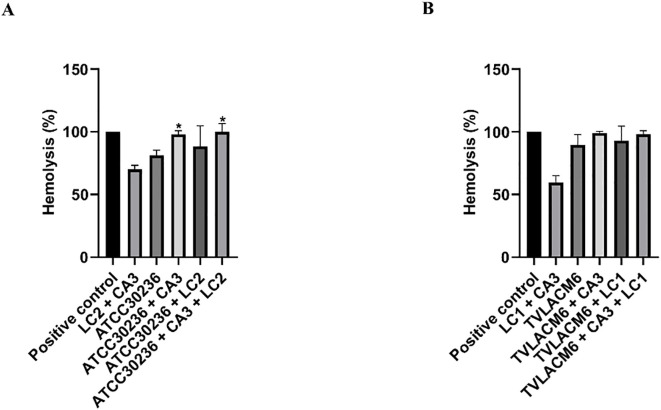
Hemolysis of erythrocytes co-incubated with monocultures or co-cultures of *Trichomonas* vaginalis, *Candida albicans* and *Lactobacillus crispatus.* **(A)** ATCC30236 *T. vaginalis* standard isolate, *C. albicans* (CA3), and *L. crispatus* (LC2). **(B)** TV-LACM6 *T. vaginalis* fresh clinical isolate, *C. albicans* (CA3), and *L. crispatus* (LC1). Positive control of hemolysis is erythrocytes treated with 0.2% Triton X-100. Results are expressed as a percentage of total hemolysis, presented as the mean ± S.D. of at least two blood samples. The percentage of hemolysis from erythrocytes co-incubated with *T. vaginalis* monocultures was compared to co-cultures with the protozoan. (*) indicates a significant difference.

The hemolytic activity of *T. vaginalis* was measured during co-incubation with erythrocytes and compared to its activity in co-culture with *C. albicans* and *L. crispatus* (**Figure 3**). Similar to its interaction with vaginal cells, the ATCC isolate showed a significant increase in hemolysis when co-cultured with *C. albicans* and in triple co-culture with erythrocytes, compared to monoculture (**Figure 3A**). Notably, the hemolytic activity of the TV-LACM6 fresh clinical isolate was higher than that of ATCC30236 (**Figure 3B**). However, unlike the ATCC isolate, co-culture did not further enhance hemolysis in the clinical isolate.

### 3.8 Inhibition of biofilm formation and yeast-to-hyphal transition in co-cultures of *T. vaginalis*, *C. albicans*, and *L. crispatus*

**Figure 4:**
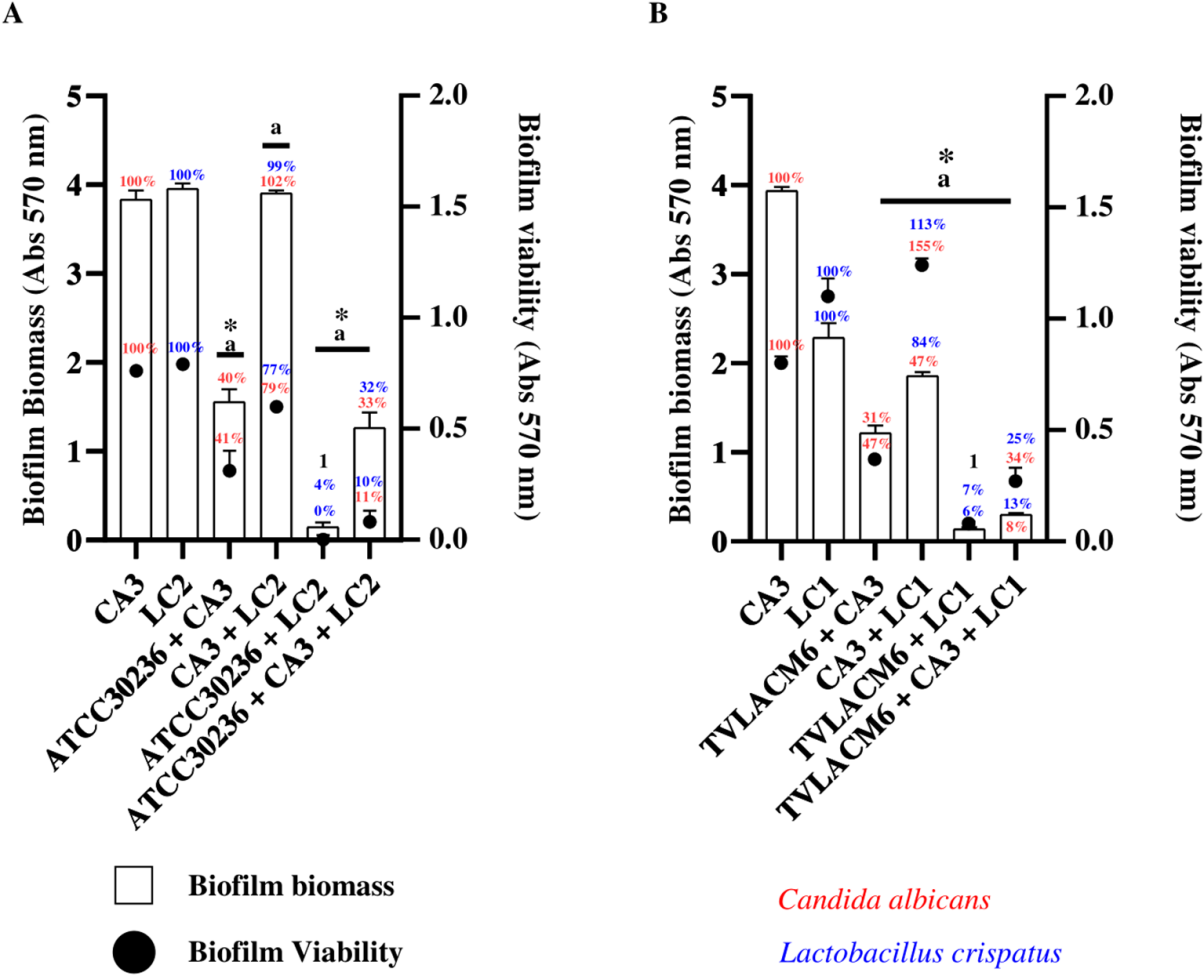
Biofilm formation and viability of *Candida albicans* and *Lactobacillus crispatus* in monoculture and co-culture **(A)** *C. albicans* (CA3, 3.33x10^4^ CFU/mL), and second initial density of *L. crispatus* (LC2, 5.53x10^6^ CFU/mL) in monoculture or co-culture. Co-culture with ATCC *T. vaginalis* isolate (ATCC30236, 1 x 10^6^ trophozoites/mL) including the combinations ATCC30236 + CA3, ATCC30236 + LC2, and ATCC30236 + CA3 + LC2. ^1^ Percentage represent biofilm biomass and biofilm viability of ATCC30236 + LC2, respectively. **(B)** Biofilm formation and viability by CA3, and first initial density of *L. crispatus* (LC1, 5.53x10^7^ CFU/mL) in monoculture or co-culture (CA3 + LC1), and in co-culture with fresh clinical *T. vaginalis* isolate (TV-LACM6) including the combinations: TV-LACM6 + CA3, TV-LACM6 + LC1, and TV-LACM6 + CA3 + LC1. (a) Statistically significant difference in biofilm viability (p < 0.05). (*) Statistically significant difference in biofilm biomass formation (p < 0.05).

The results of biofilm biomass and metabolic viability assessments are presented in **Figure 4**. Biofilm biomass after 24 hours of incubation was evaluated in monocultures of *C. albicans* (CA3, initial density 3.3x10^4^ CFU/mL) and *L. crispatus* (LC2, initial density 5.53x10^6^ CFU/mL), which were set as the baseline (100%).

For *C. albicans* biofilm biomass, co-culture with the ATCC *T. vaginalis* isolate (ATCC30236, initial density 1.00x10^6^ trophozoites/mL) led to a 60% reduction, with biofilm decreasing to 40%).A more pronounced reduction (67%) was observed in the triple co-culture (ATCC30236, CA3, and LC2), resulting in a biofilm biomass of 33%).In the case of *L. crispatus*, co-culture with ATCC30236 led to a 96% reduction in total biofilm biomass (remaining biomass 4.0%), while the triple co-culture reduced biomass by 68% (remaining biomass 32%). Notably, the co-culture of CA3 and LC2 alone did not significantly impact biofilm biomass, which remained close to 100%, indicating that the presence of *T. vaginalis* played a crucial role in biofilm disruption (**Figure 4A**).

Biofilm metabolic viability was also assessed, expressed as a percentage relative to biofilm viability of the monocultures of CA3 and LC2, which were set as the baseline (100%). For *C. albicans* (CA3), co-culture with LC2 reduced viability by 21% (remaining viability 79%), When co-cultured with ATCC30236, metabolic viability was further reduced by 59% (remaining viability 41%), and in the triple co-culture (ATCC30236, CA3, and LC2), viability was inhibited by 89% (remaining viability 11%). In the case of *L. crispatus* (LC2), co-culture with ATCC30236 completely abolished biofilm viability (0%), while co-culture with CA3 resulted in a 33% reduction (remaining viability 77%). The triple co-culture significantly inhibited biofilm viability by 90% (remaining viability 10%) (**Figure 4A**).

A similar trend was observed when biofilm biomass and viability were evaluated using the fresh clinical *T. vaginalis* isolate (TV-LACM6). For *C. albicans* (CA3)biofilm biomass, co-culture with the TV-LACM6resulted in a 69% reduction (remaining biofilm biomass 31%). When co-cultured with LC1biofilmbiomass was reduced by 53% (remaining biomass 47%), while the co-culture (TV-LACM6,CA3, and LC1) resulted in a dramatic a 92% reduction (remaining biomass 8%).In the case of *L. crispatus* (LC1), co-culture with TV-LACM6 led to a 94% reduction in biofilm biomass (remaining biomass 6%), while co-culture with CA3 and LC1 resulted in a more moderate 16% reduction (remaining biomass 84%). The triple co-culture led to an 87% reduction in biofilm biomass (remaining biomass 13%) (**Figure 4B**).

Regarding metabolic biofilm viability co-culture with LC1 significantly increased *C. albicans* viability to 155%, suggesting a protective effect However, in the presence of TV-LACM6, biofilm viability was reduced by 53% (remaining viability 47%),and the triple co-culture (TV-LACM6, CA3, and LC1) resulted in a 66% reduction (remaining viability34%). *L. crispatus* (LC1) biofilm viability also increased in co-culture with CA3 (113%), while co-culture with TV-LACM6 significantly reduced biofilm viability by 93% (remaining viability 7%.The triple co-culture inhibited viability by 75% (remaining viability 25%).

**Figure 5:**
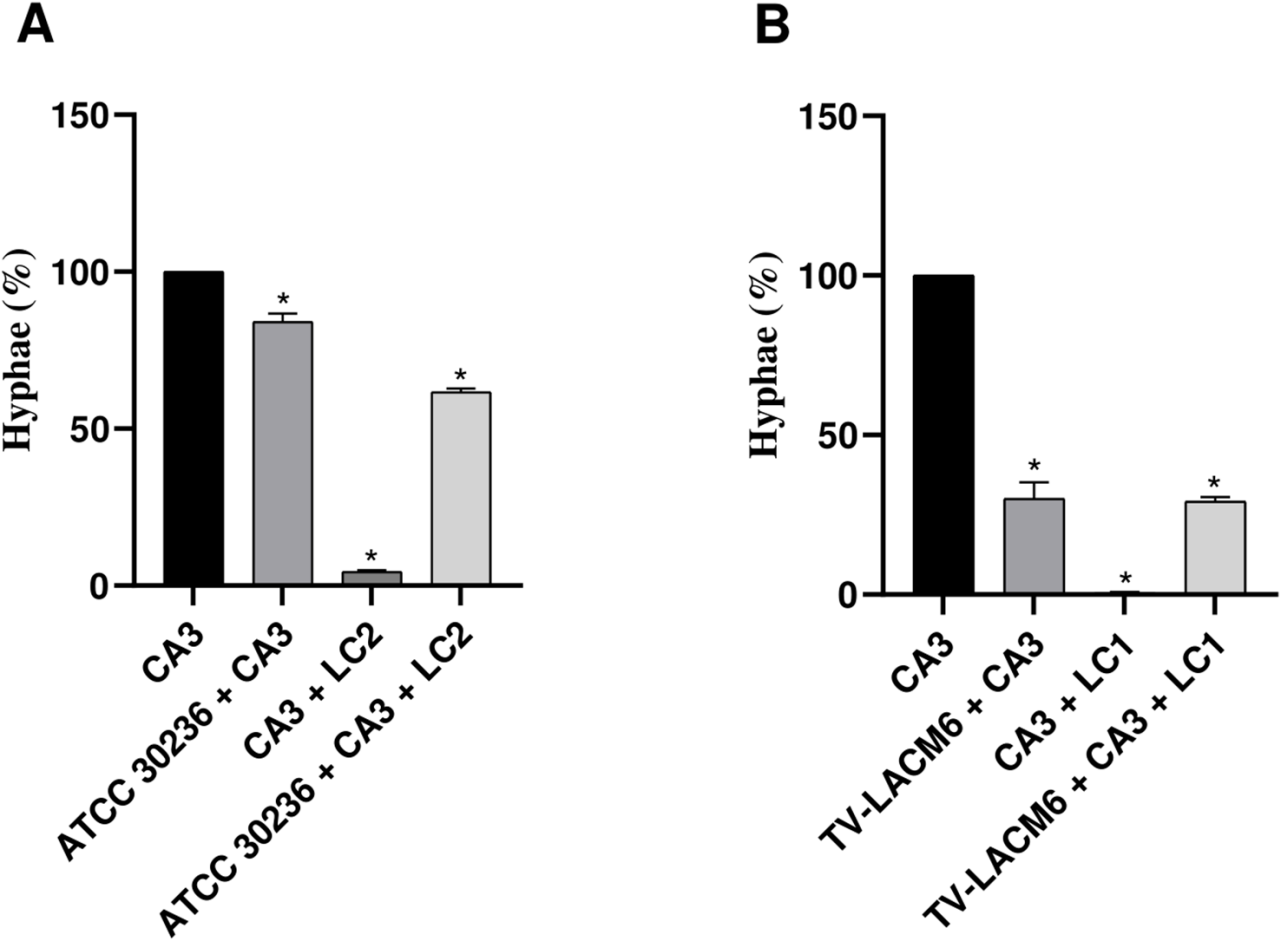
Yeast-to-hyphal form transition of *Candida albicans* in monoculture and co-culture. **(A)** Co-culture of *C. albicans* (CA3, initial density at 3.33x10^4^ CFU/mL) with ATCC *Trichomonas vaginalis* isolate (ATCC3026, initial density at 1 x 10^6^ trophozoites/mL) and second density of *Lactobacillus crispatus* (LC2, *i*nitial density at 5.53x10^6^ CFU/mL). **(B)** Co-culture of CA3 with fresh clinical *T. vaginalis* isolate (TV-LACM6, initial density at 1 x 10^6^ trophozoites/mL) and first density of *L. crispatus* (LC1, initial density at 5.53x10^7^ CFU/mL. The date were expressed by percentage de hyphae formation. Results are representative of two independent experiments conducted with triplicate assays. (*) Statistically significant difference (p < 0.05).

Additionally, the yeast-to-hyphal transition of *C. albicans* (CA3) was quantified by microscopy, with yeast and hyphal forms expressed as percentages of the total observed forms (set as 100%) (**Figure 5**). When CA3 was co-cultured with ATCC 30236, the yeast-to-hyphal transition rate was 84.1%, indicating an inhibition of 15.9%. The presence of *L. crispatus* (LC2) significantly reduced the transition rate to 4.41%, representing 95.6% inhibition. In the triple co-culture (ATCC30236, CA3, and LC2), the transition rate was 38.28%, reflecting an inhibition of 61.71% (**Figure 5A**).

A stronger inhibition effect was observed with the fresh clinical isolate (TV-LACM6). The yeast-to-hyphal transition rate in co-culture with TV-LACM6 was 30.09%, representing to 71.91% inhibition. Co-culture of LC1 with CA3 nearly abolished hyphal formation, with a transition rate of 0.63%, indicating 99.37% inhibition. The triple co-culture (TV-LACM6, CA3, and LC1) resulted in a transition rate of 29.23%, corresponding to 70.77% inhibition rate.

### 3.9 Morphological analysis of *T.vaginalis*, *C. albicans*, and *L. crispatus* in co-culture using Scanning Electron Microscopy

**Figure 6:**
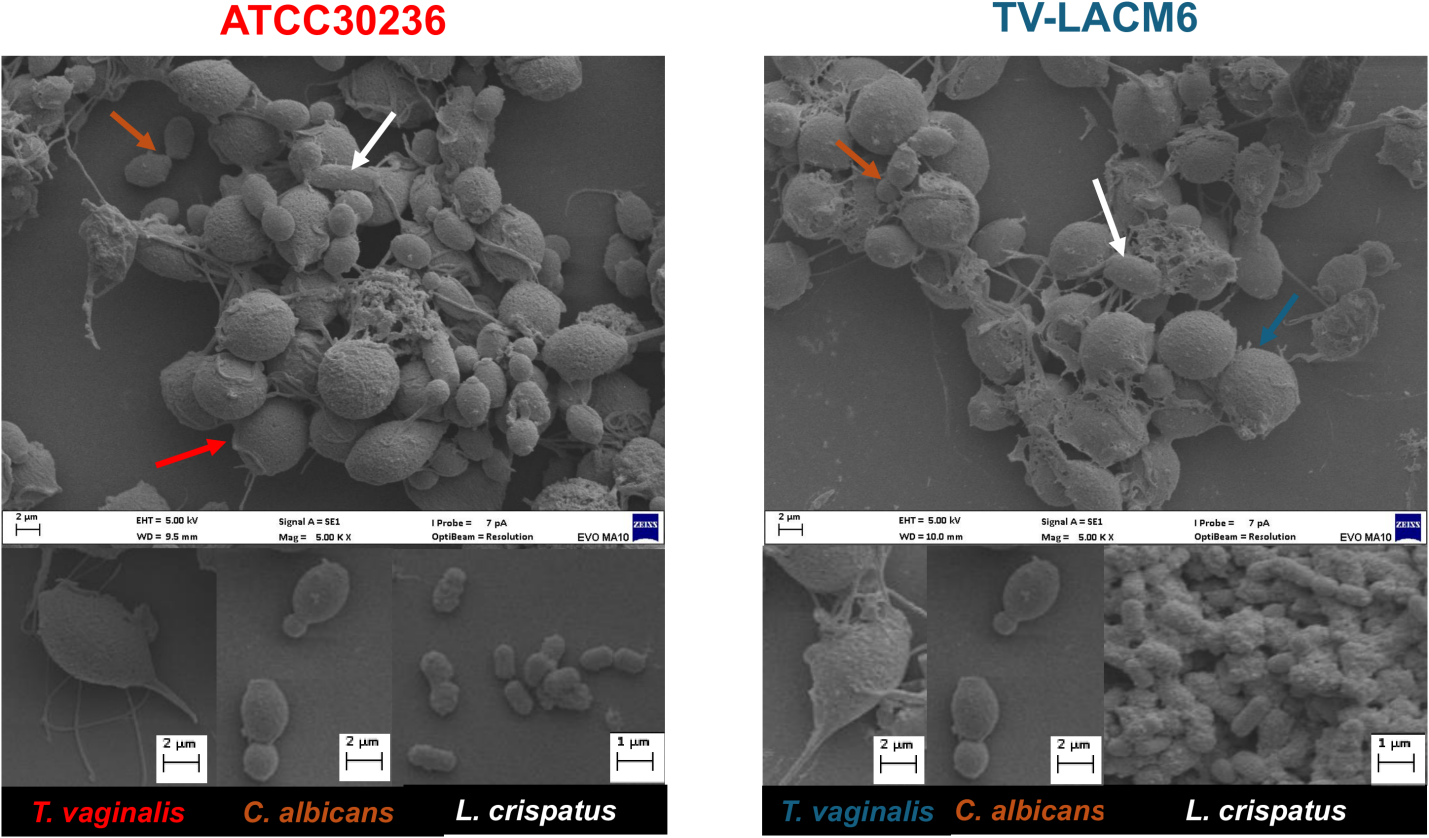
Scanning electron microscopy of ATCC 30236 and TV-LACM6 isolates in monoculture or co-culture with ATCC 24433 C. *albicans* and CCT 7595 *L. crispatus.* Left side: Co-culture of the *T. vaginalis* ATCC30236 isolate (initial density 1.00x10^6^ trophozoites/mL, red arrow), *C albicans* CA3 (initial density 3.3x10^4^ CFU/mL, orange arrow) and *L. crispatus* LC2 (initial density 5.53x10^6^ CFU/mL, white arrow). Right side: Co-culture of fresh clinical isolate TV-LACM6 (initial density 1.00x10^6^ trophozoites/mL, blue arrow), *C albicans* CA3 (orange arrow), and *L. crispatus* LC2 (initial density 5.53x10^6^ CFU/mL, white arrow).

The morphology of *T.* vaginalis, *C. albicans*, and *L. crispatus* was analyzed using SEM in both monoculture and co-culture conditions. The *T. vaginalis* isolates ATCC30236 and TV-LACM6 were co-cultured with *C. albicans* (CA3) and *L. crispatus* (LC1 or LC2), under pre-selected conditions, and their morphology was evaluated after incubation. **Figure 6A** depicts the co-culture of the *T. vaginalis* ATCC30236 isolate (initial density, 1.00x10^6^ trophozoites/mL, marked with a red arrow), *C albicans* CA3 (3.3x10^4^ CFU/mL, orange arrow) and *L. crispatus* LC2 (5.53x10^6^ CFU/mL, white arrow). Similarly, **Figure 6B**, shows the co-culture of the fresh clinical isolate TV-LACM6 (1.00x10^6^ trophozoites/mL, blue arrow) with *C albicans* CA3 (orange arrow), and *L. crispatus* LC1 (5.53x10^7^ CFU/mL, white arrow).

The SEM micrographs revealed differences in trophozoite morphology between the isolates. In the triple co-culture on MRS medium, the ATCC30236 isolate exhibited a body length of 8.6 µm and width of 5.3 µm. In contrast, trophozoites of the fresh clinical isolate TV-LACM6 were smaller, measuring 6.1 µm in length and 4.33 µm in width. Regarding the bacterial and fungal counterparts, *C. albicans* ATCC 24433 was observed exclusively in yeast form, with an average length of 3.98 µm and width of 2.6 µm. Meanwhile, *L. crispatus* CCT 7595 measured 4.8 µm in length and 2.3 µm in width. These observations highlight morphological variations between the *T. vaginalis* isolates and confirm the distinct structural characteristics of *C. albicans* and *L. crispatus* under co-culture conditions.

## 4 Discussion

The flagellated protozoan *T. vaginalis* causes trichomoniasis, a sexually transmitted infection characterized by a dysbiotic microbiome primarily composed of diverse anaerobic bacteria. These bacteria can function as pathobionts, exacerbating the pathogenic effects of the protozoan (Hinderfeld and Simoes-Barbosa, 2020). During its establishment and proliferation in the vagina, *T. vaginalis* employs mechanisms that damage Lactobacillus species, including phagocytosis, which leads to decreased densities of these protective bacteria in the vaginal environment. Additionally, trichomoniasis is associated with an increase in vaginal pH (Kalia et al., 2020). These same conditions are also related to vulvovaginal candidiasis (VVC), a vaginal infection caused by the opportunistic pathogen *Candida* spp., with *C. albicans* being the predominant species. VVC is linked to imbalances in the surrounding microbiota, inflammatory processes, the use of broad-spectrum antibiotics, and states of immunodeficiency (Brown et al., 2012; Nyirjesy et al., 2022).

In this study, we standardized a co-culture of *T. vaginalis*, *C. albicans*, and *L. crispatus* to establish a new platform for evaluating the potential of synthesized compounds or natural products as novel drugs for the treatment of trichomoniasis and VVC. This co-culture provides an experimental microenvironment where these microorganisms can interact and compete for nutrients available in the medium. We selected MRS medium for co-culture due to its suitability for the growth of *Lactobacillus* species. This medium is rich in essential nutrients for bacterial development, including polysorbate, acetate, magnesium, and manganese (De Man et al., 1960). To support the growth of *T. vaginalis* in this medium, we added adult bovine serum. Serum is also used as a supplement in TYM, the standard culture medium for *T. vaginalis*, and it provides essential molecules for trophozoite survival, such as adenosine (Frasson et al., 2012). For *C. albicans*, serum acts as an inducer of hyphae development, containing glycoprotein-derived substances such as acetylglucosamine and proline, which are associated with the transition from yeast to hyphal forms (Villa et al., 2020).

During the standardization of initial densities for each microorganism, we observed that high densities of *L. crispatus* inhibited the growth of the *T. vaginalis* ATCC 30236 isolate and reduced the growth of *C. albicans*. This inhibition can be attributed to the higher density of *L. crispatus* relative to the other microorganisms, providing a competitive advantage for nutrient acquisition during the 24-hour incubation period. Additionally, *L. crispatus* produces antimicrobial substances, such as bacteriocins and hydrogen peroxide (Kalia et al., 2020; Scillato et al., 2021). Furthermore, *Lactobacillus* species ferment carbohydrates to produce lactic acid, resulting in an acidic environment. In our results, co-cultures containing *L. crispatus* at the highest initial density (LC1, 5.53 x 10^7^ CFU/mL) with *T. vaginalis* and/or *C. albicans* yielded a pH of approximately 4.0 after 24 hours of incubation. Previous studies indicate that the predominance of *Lactobacillus* in the vaginal microbiota typically results in an acidic pH of around 3.5 (O’Hanlon et al., 2013).

Petrin et al. (1998) demonstrated that *T. vaginalis* can thrive at a pH close to 4.5. However, following the proliferation of the parasite, the pH increases, leading to the phagocytosis of *Lactobacillus* species. In the selected combination for the triple co-culture, *T. vaginalis* ATCC isolate (TV1, ATCC30236) was introduced at an initial density of 1.00 x 10^6^ trophozoites/mL, *C. albicans* at 3.3x10^4^ CFU/mL (CA3), and *L. crispatus* at 5.53x10^6^ CFU/mL. After 24 hours of incubation, all microorganisms exhibited higher densities than at the beginning of the experiment, with the pH measured at 5.0. This observation suggests a balance between nutrient competition and the mechanisms that promote the proliferation of specific microorganisms, as evidenced by the growth kinetics.

A study demonstrated that the vaginal fluid of healthy women predominantly contains *Lactobacillus* spp. at a density of 10^7^ to 10^8^ CFU/mL (Andreu et al., 1995). Lehker and Alderete (1990) found that *T. vaginalis* isolates can maintain higher densities (1 x 10^6^ trophozoites/mL to 1 x 10^7^ trophozoites/mL) in a medium containing trypticase-yeast extract-maltose (TYM) supplemented with inactivated horse serum. Similarly, densities comparable to these were achieved in the co-culture of *T. vaginalis*, *C. albicans*, and *L. crispatus* in MRS medium. The ATCC30236 *T. vaginalis* isolate exhibited a mean density of 2.4 x 10^6^ trophozoites/mL after 24 hours, while *C. albicans* showed a mean density of 2.6 x 10^6^ CFU/mL, and *L. crispatus* demonstrated a mean density of 7.9 x 10^6^ CFU/mL after the same incubation period. Notably, the transition of *C. albicans* from yeast to hyphae is stimulated by low Candida cell densities, specifically below 10^7^ cells/mL (Mayer et al., 2013).

On the other hand, during the standardization of the co-culture using the fresh clinical *T. vaginalis* TV-LACM6 isolate at the same initial density as the ATCC30236 isolate, *C. albicans* was set at an initial density of 3.3 x 10^4^ CFU/mL (the same density used in co-culture with the ATCC isolate). In contrast, *L. crispatus* varied in the first two initial densities (LC1: 5.53 x 10^7^ CFU/mL and LC2: 5.53 x 10^6^ CFU/mL). Unlike the ATCC isolate, the growth of the TV-LACM6 isolate was unaffected by the two highest initial densities of *L. crispatus*. Consequently, the optimal combination was determined to be TV-LACM6 at an initial density of 1.00 x 10^6^ trophozoites/mL, *C. albicans* at 3.3 x 10^4^ CFU/mL (CA3), and *L. crispatus* at 5.53 x 10^7^ CFU/mL (LC1). Previous studies by our group indicated that TV-LACM6 exhibited greater resistance to metronidazole (da Luz Becker et al., 2015) and was the most cytolytic isolate against vaginal epithelial cells, showing low ecto-5’-nucleotidase activity, an enzyme that hydrolyzes AMP to adenosine. This low enzymatic activity in the TV-LACM6 isolate contributes to a reduced amount of extracellular adenosine, which has a protective effect against vaginal cellular damage (Menezes et al., 2017).

The cytotoxic effect was confirmed through LDH release and hemolysis assays, with the fresh clinical isolate demonstrating a more pronounced effect compared to ATCC30236. However, when ATCC30236 was co-cultured with *C. albicans* and *L. crispatus*, an increase in LDH release and hemolysis was observed. This difference between monoculture and co-culture was not evident when TV-LACM6 was in co-culture. This disparity can be attributed to the varying initial densities of *L. crispatus* used in the co-culture with ATCC30236 (LC2) and TV-LACM6 (LC1). Additionally, variations in cytolysis and hemolysis have been observed between fresh clinical and ATCC *T. vaginalis* isolates (Menezes et al., 2017). Fresh clinical isolates are adapted to significant fluctuations in the medium, such as nutrient supply, oxygen levels, and pH, due to their exposure to other microorganisms and environmental factors at the infection site. In contrast, the ATCC *T. vaginalis* isolate is a long-term cultured organism adapted to a stable *in vitro* environment, leading to expectations of overexpression of virulence factors when challenged. These findings suggest that distinct biological processes at both transcriptional and translational levels may be employed by trophozoites during host cell colonization, with varying nutrient requirements in this metabolic context (Lehker and Alderete, 1990; Arroyo et al., 1992; Menezes et al., 2016).

Trichomoniasis and vulvovaginal candidiasis (VVC) are frequently associated with treatment-resistant isolates, leading to significant public health costs. Consequently, the discovery of alternative drugs is crucial. *In vitro* tests that accurately mimic real infection conditions in the host and detect antimicrobial activity are of paramount importance. The drug discovery and development process typically spans 12 to 15 years and exceeds 1 billion dollars, with basic research playing a vital role in target identification and molecule selection (Hughes et al., 2010). Translational research aims to bridge the gap between basic science findings and human studies (Rubio et al., 2010). However, there exists a “Death Valley” between basic research and clinical drug development, which hampers translational efforts. Furthermore, neglected and widespread infections present significant challenges for translational research (Rigo et al., 2022).

In this context, co-culture systems with carefully optimized initial microorganism densities provide a valuable tool for evaluating the antimicrobial activity of drugs in a more physiologically relevant environment. Our results revealed that the minimum inhibitory concentration (MIC) of metronidazole against *T. vaginalis* isolates was lower in co-culture compared to monoculture. This reduction suggests that the presence *L. crispatus* plays a significant role in enhancing the drug’s efficacy. Notably, a more pronounced effect was observed with the *T. vaginalis* isolate TV-LACM6 isolate, which was co-cultured with *L. crispatus* at a higher initial density (LC1).

The interaction between *T. vaginalis* and lactobacilli has been previously described. Fichorova et al. (2013) reported that *T. vaginalis* exhibited antagonistic interactions with *L. acidophilus*, *L. crispatus*, and *L. jensenii*, while demonstrating synergy with bacterial species associated with bacterial vaginosis. This suggests that the microbial composition of the environment can significantly influence *T. vaginalis* behavior and drug susceptibility. A similar trend was observed in the case of *C. albicans* treated with fluconazole. The minimum fungicidal concentration (MFC) of fluconazole was reduced in co-culture, likely due to the antimicrobial compounds secreted by *L. crispatus* and the ability of *T. vaginalis* to engulf yeast cells. The latter mechanism, described by Pereira-Neves and Benchimol (2007), is mediated by a mannose receptor on the parasite’s surface, facilitating direct interactions with yeast. These findings have important implications for translational research, as co-culture system more closely mimic the complexity of *in vivo* microbial communities compared to traditional monocultures systems. Beyond the direct effects of antimicrobial agents, the microenvironment itself plays a crucial role in modulating pathogen survival and drug efficacy. This highlights the necessity of considering microbial interactions when developing treatment strategies for polymicrobial infections.

*C. albicans* is capable of growth under acidic pH conditions, with the yeast form predominantly supporting this, as we observed the presence of the yeast form in co-culture, even with the addition of bovine serum. The inhibition of the yeast-to-hyphal transition, biofilm biomass, and biofilm viability of *C. albicans* can be attributed to substances produced by *L. crispatus* and the presence of *T. vaginalis*. Studies have indicated that *Lactobacillus* spp. can inhibit the transition from yeast to hyphae and compete with fungal cells for mucosal adhesion (Sun et al., 2023). Therefore, our hypothesis is that the biomass and viability detected on combination of *C. albicans* and *L. crispatus* is referent as biofilm formed by bacteria. However, studies utilizing molecular biology and scanning electronic microscopy are necessary for confirmation. *T. vaginalis* also affects these factors, with more pronounced inhibition observed when TV-LACM6 was co-cultured. These findings suggest that the fresh clinical isolate has a more pronounced phagocytic activity, reducing the number of fungi cells. Studies have shown that *T. vaginalis* is capable of phagocytizing *Lactobacillus* spp., vaginal epithelial cells, leukocytes, erythrocytes, sperm cells, *Neiseseria gonorrhoea*, and other bacteria such as *Staphylococcus aureus* and *Pseudomonas aeruginosa* (Midlej and Benchimol, 2010). In **Figure 6B** it is observed smaller numbers of yeast-like structures and bacteria. Additionally, in the lower part of the image, a trophozoite with an amoeboid structure was visible, potential exhibiting phagocytic activity as it appeared to internalize other microorganisms. These observations support the previously noted pronounced phagocytic activity of *T. vaginalis* (Pereira-Neves and Benchimol, 2007).

The size of microorganisms is influenced by physicochemical conditions such as temperature, pH, and nutrient availability. In this context, fresh isolates of *T. vaginalis* typically display shorter body lengths and widths compared to cultured trophozoites, measuring 8.5 µm and 5.7 µm, respectively, versus 9.5 µm and 6.8 µm for cultured forms (Cheon et al., 2013). This was demonstrated in the SEM analysis by comparing the size of the ATCC isolate (8.6 µm in length and 5.3 µm in width) to that of the TV-LACM6 fresh clinical isolate (6.1 µm in length and 4.3 µm in width). However, it is noteworthy that the size of the ATCC isolate was shorter than the values reported in literature. In SEM analysis, *C. albicans* grown on YPD agar exhibited yeast form measuring 3.8 µm in length and 2.7 µm in width (Staniszewska et al., 2013). In our findings, the size of the yeast forms did not change significantly when grown on MRS medium in co-culture, measuring 3.98 µm in length and 2.6 µm in width. For *L. crispatus*, the size of the bacteria in monoculture was 4.3 µm in length and 1.6 µm in width. In co-culture, the size showed no pronounced modification, measuring 4.8 in length and 2.3 µm in width.

## 5 Conclusion

In summary, our findings suggest that *in vitro Trichomonas vaginalis, Candida albicans* and *Lactobacillus crispatus* co-culture represents a valuable method for assessing the antimicrobial efficacy of novel compounds against pathogens implicated in vaginitis. The substances generated by *L. crispatus* in our standardized co-culture model offer potential avenues for further investigation. This study underscores the utility of co-culture systems in elucidating complex microbe-microbe and drug-microbe interactions, providing valuable insights for translational research and future therapeutic development.

## Data Availability

The original contributions presented in the study are included in the article/**Supplementary Material**. Further inquiries can be directed to the corresponding author.
