# Correction: *In vitro* co-culture model of *Trichomonas vaginalis*, *Candida albicans*, and *Lactobacillus crispatus*: a system for assessing antimicrobial activity and microorganism interactions in vaginitis

**DOI:** 10.3389/fpara.2025.1711595

**Published:** 2025-10-10

**Authors:** Fernanda Gomes Cardoso, Luisa Trindade dos Santos, Saulo Almeida Menezes, Graziela Vargas Rigo, Tiana Tasca

**Affiliations:** Faculdade de Farmácia and Centro de Biotecnologia, Universidade Federal do Rio Grande do Sul, Porto Alegre, RS, Brazil

**Keywords:** *Candida albicans*, co-culture, *Lactobacillus crispatus*, *Trichomonas vaginalis*, vaginal microbiota, vaginitis

In the published article, there was a mistake in **Figure 3** and **Figure 5**. **Figure 3** appeared in place of **Figure 5** and vice versa. The corrected figures appear below.

**Figure 3:**
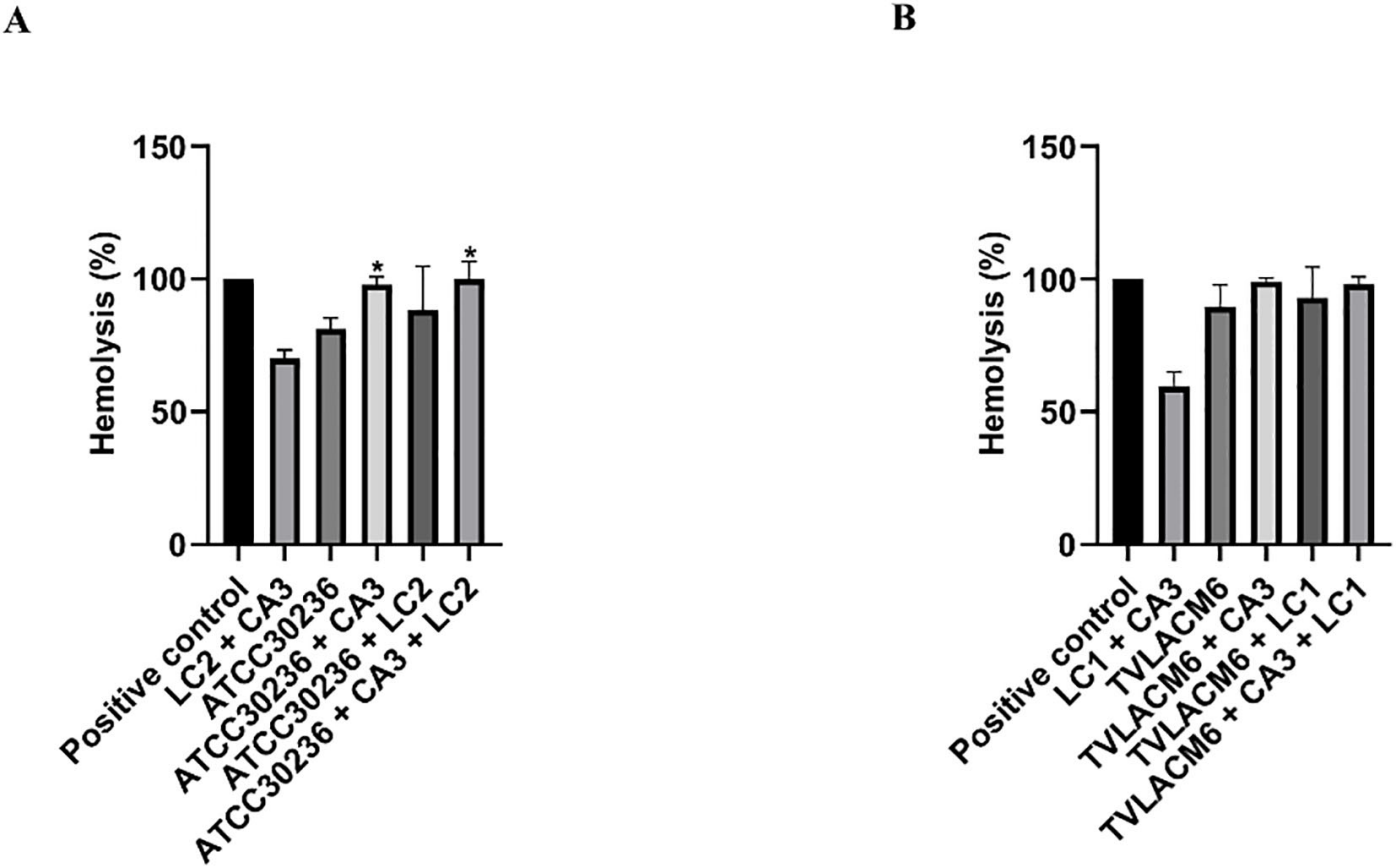
Hemolysis of erythrocytes co-incubated with monocultures or co-cultures of *Trichomonas* vaginalis, *Candida albicans* and *Lactobacillus crispatus.* **(A)** ATCC30236 *T. vaginalis* standard isolate, *C*. *albicans* (CA3), and *L. crispatus* (LC2). **(B)** TV-LACM6 *T. vaginalis* fresh clinical isolate, *C*. *albicans* (CA3), and *L. crispatus* (LC1). Positive control of hemolysis is erythrocytes treated with 0.2% Triton X-100. Results are expressed as a percentage of total hemolysis, presented as the mean ± S.D. of at least two blood samples. The percentage of hemolysis from erythrocytes co-incubated with *T. vaginalis* monocultures was compared to co-cultures with the protozoan. (*) indicates a significant difference.

**Figure 5:**
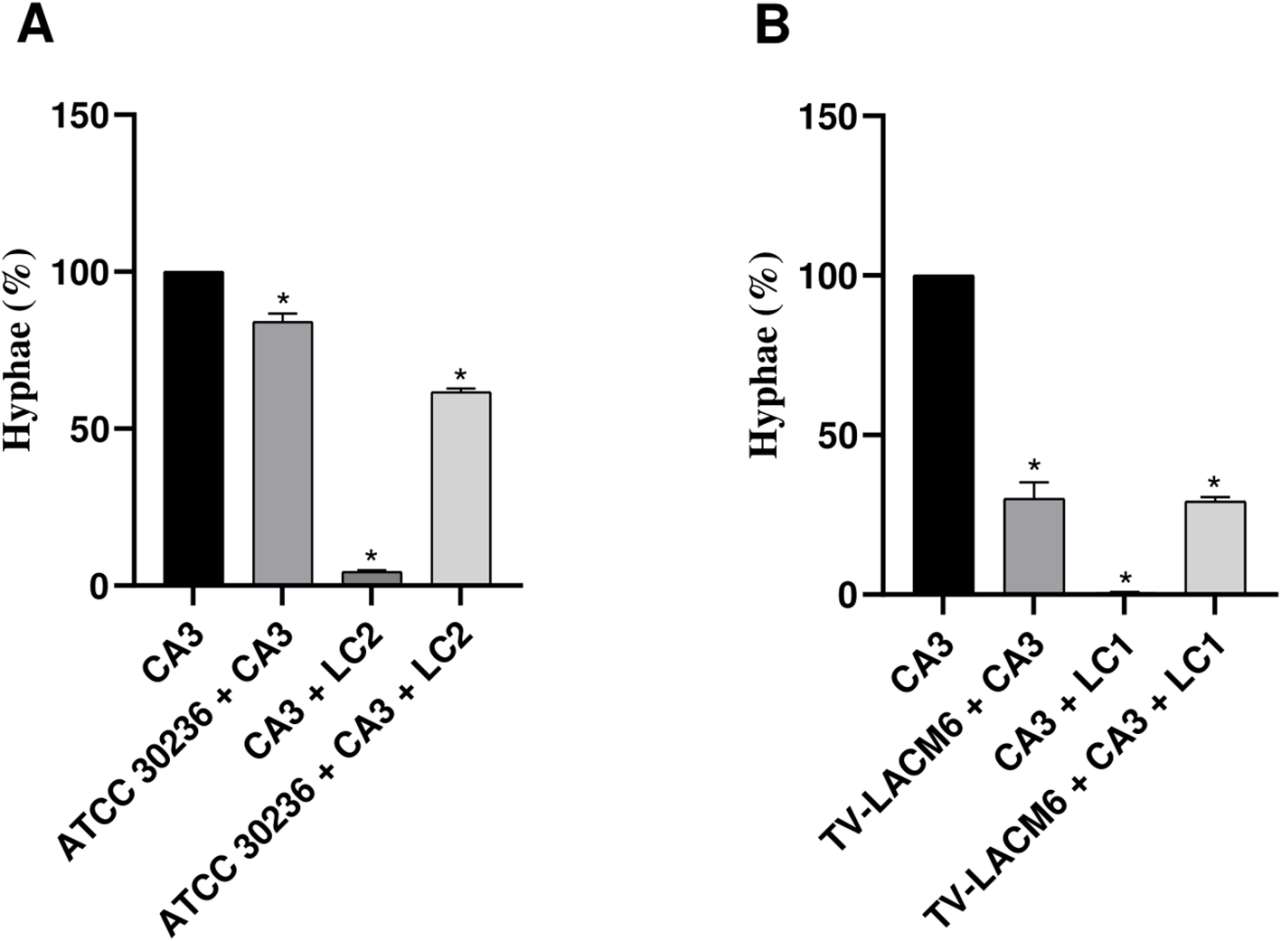
Yeast-to-hyphal form transition of *Candida albicans* in monoculture and co-culture. **(A)** Co-culture of *C*. *albicans* (CA3, initial density at 3.33x10^4^ CFU/mL) with ATCC *Trichomonas vaginalis* isolate (ATCC3026, initial density at 1 x 10^6^ trophozoites/mL) and second density of *Lactobacillus crispatus* (LC2, *i*nitial density at 5.53x10^6^ CFU/mL). **(B)** Co-culture of CA3 with fresh clinical *T. vaginalis* isolate (TV-LACM6, initial density at 1 x 10^6^ trophozoites/mL) and first density of *L. crispatus* (LC1, initial density at 5.53x10^7^ CFU/mL. The date were expressed by percentage de hyphae formation. Results are representative of two independent experiments conducted with triplicate assays. (*) Statistically significant difference (p < 0.05).

The original article has been updated.

