# Supplementary material for: In vitro co-culture model of Trichomonas vaginalis, Candida albicans, and Lactobacillus crispatus: a system for assessing antimicrobial activity and microorganism interactions in vaginitis
Source: Front Parasitol. 2025 Apr 14;4:1523113. doi: 10.3389/fpara.2025.1523113 (PMC12034676; doi:10.3389/fpara.2025.1523113)
Supplement: Supplementary file 1 [file DataSheet1.pdf]

frontiers

Supplementary Material

Supplementary Table 1. Growth Status of *Trichomonas vaginalis*, *Candida albicans* and *Lactobacillus crispatus* in Four Culture Media.

**Medium**	***T. vaginalis***	***C. albicans***	***L. crispatus***	**Vaginal cell**
**TYM + 10% ABS**	Presence	Presence	Absence	Not applicable
**SDB + 10% ABS**	Presence	Presence	Absence	Not applicable
**MRS + 10% ABS**	Presence	Presence	Presence	Not applicable
**MRS + 10% ABS + 20 µM FLZ**	Presence	Absence	Presence	Not applicable
**RPMI + 20% FBS**	Presence	Presence	Presence	Presence

TYM: trypticase-yeast extract-maltose; ABS: adult bovine serum; SDB: Sabouraud dextrose broth; FLZ: fluconazole; FBS: fetal bovine serum.

Supplementary Table 2. Summary of all microorganism combinations (monoculture or co-culture) in the chequerboard assays with initial density and mean trophozoites/mL ± SD of *Trichomonas vaginalis* (TV) and mean CFU/mL ± SD of *Candida albicans* (CA) and *Lactobacillus crispatus* (LC) after 24 h.

**Fixed MO**	**Condition Or Combination**	**Initial density (10^5) Trophozoites/mL[#] CFU/mL[##]**	**After 24 h**		
			**Trophozoites/mL (10^5) ± SD**	***C. albicans* CFU/mL (10^5) ± SD**	***L. crispatus* CFU/mL (10^5) ± SD**
**TV1**	**TV1**	10.0[#]	**30.37 ± 2.32**	-	-
**LC2**			**26.80 ± 2.52**	-	-
**CA3**			**29.75 ± 3.64**	-	-
**LC2**	TV2	1.00[#]	4.03 ± 0.75	-	-
**CA3**			4.80 ± 1.08	-	-
**TV1**	LC1	553.00[##]	-	-	101.00 ± 2.12
**CA3**			-	-	97.50 ± 6.36
**TV1**	**LC2**	55.30[##]	-	-	87.30 ± 1.06
**CA3**			-	-	81.30 ± 1.77
**LC2**			-	-	88.80 ± 3.18
**TV1**	LC3	5.53[##]	-	-	76.50 ± 0.70
**CA3**			-	-	74.45 ± 6.36
**TV1**	LC4	0.55[##]	-	-	70.80 ± 0.35
**CA3**			-	-	60.50 ± 9.19
**TV1**	LC5	0.05[##]	-	-	65.00 ± 3.54
**CA3**			-	-	52.50 ± 6.36
**TV1**	CA1	33.30[##]	-	54.00 ± 0.00	-

**LC2**			-	52.00 ± 3.54	-
**TV1**	CA2	3.33$^{\#\#}$	-	44.80 ± 1.05	-
**LC2**			-	42.00 ± 4.20	-
**TV1**	**CA3**	0.33$^{\#\#}$	-	**35.50 ± 0.70**	-
**CA3**			-	**42.00 ± 1.41**	-
**LC2**			-	**37.30 ± 2.47**	-
**TV1**	CA4	0.03$^{\#\#}$	-	30.50 ± 1.41	-
**LC2**			-	33.80 ± 1.06	-
**TV1**	CA5	0.003$^{\#\#}$	-	27.80 ± 1.06	-
**LC2**			-	33.00 ± 2.12	-
**TV1**	TV1 + LC1	10.00$^{\#}$ + 553.00$^{\#\#}$	0.00 ± 0.00*	-	70.50 ± 4.24*
**TV1**	**TV1 + LC2**	10.00$^{\#}$ + 55.30$^{\#\#}$	**13.05 ± 3.61***	-	**65.30 ± 8.13**
**LC2**			**17.73 ± 1.42***	-	**57.50 ± 0.71***
**LC2**	TV2 + LC2	1.00$^{\# +}$ + 55.30$^{\#\#}$	0.00 ± 0.00*	-	81.30 ± 1.77
**TV1**	TV1 + LC3	10.00$^{\#}$ + 5.53$^{\#\#}$	23.17 ± 2.00*	-	59.00 ± 0.71*
**TV1**	TV1 + LC4	10.00$^{\#}$ + 0.55$^{\#\#}$	27.40 ± 2.52	-	55.50 ± 2.12*
**TV1**	TV1 + LC5	10.00$^{\#}$ + 0.05$^{\#\#}$	28.57 ± 2.06	-	40.00 ± 6.01*
**TV1**	TV1 + CA1	10.00$^{\#}$ + 33.30$^{\#\#}$	21.60 ± 0.14*	42.30 ± 3.18	-
**TV1**	TV1 + CA2	10.00$^{\#}$ + 3.33$^{\#\#}$	26.30 ± 2.26	40.00 ± 1.41	-
**TV1**	**TV1 + CA3**	10.00$^{\#}$ + 0.33$^{\#\#}$	**31.40 ± 2.97**	**39.00 ± 2.83**	-
**CA3**			**32.20 ± 0.57**	**41.00 ± 0.71**	-
**CA3**	TV2 + CA3	1.00$^{\#}$ + 5.53$^{\#\#}$	5.50 ± 0.14	36.50 ± 4.24	-
**TV1**	TV1 + CA4	10.00$^{\#}$ + 0.03$^{\#\#}$	28.75 ± 3.04	39.50 ± 4.95	-
**TV1**	TV1 + CA5	10.00$^{\#}$ + 0.003$^{\#\#}$	30.30 ± 3.97	17.00 ± 2.83*	-
**LC2**	LC2 + CA1	55.30$^{\#\#}$ + 33.30$^{\#\#}$	-	37.30 ± 2.47*	79.50 ± 0.00
**LC2**	LC2 + CA2	55.30$^{\#\#}$ + 3.33$^{\#\#}$	-	21.50 ± 2.12*	76.50 ± 1.41*
**LC2**	**LC2 + CA3**	55.30$^{\#\#}$ + 0.33$^{\#\#}$	-	**1.50 ± 0.71***	**79.30 ± 4.60**
**CA3**			-	**8.25 ± 3.54***	**62.00 ± 2.83***
**LC2**	LC2 + CA4	55.30$^{\#\#}$ + 0.03$^{\#\#}$	-	0.25 ± 0.35*	78.30 ± 1.77
**LC2**	LC2 + CA5	55.30$^{\#\#}$ + 0.003$^{\#\#}$	-	0.00 ± 0.00*	80.00 ± 0.00
**CA3**	CA3 + LC1	553.00$^{\#\#}$ + 0.33$^{\#\#}$	-	3.75 ± 2.47*	61.80 ± 2.47*
**CA3**	CA3 + LC3	5.53$^{\#\#}$ + 0.33$^{\#\#}$	-	32.30 ± 3.18*	63.30 ± 4.60
**CA3**	CA3 + LC4	0.55$^{\#\#}$ + 0.33$^{\#\#}$	-	34.80 ± 1.77*	61.80 ± 2.47
**CA3**	CA3 + LC5	0.05$^{\#\#}$ + 0.33$^{\#\#}$	-	39.00 ± 1.41	55.00 ± 7.07
**TV1**	TV1 + CA1 + LC1	10.00$^{\#}$ + 33.30$^{\#\#}$ + 553.00$^{\#\#}$	3.50 ± 2.43*	50.00 ± 2.83	85.30 ± 1.06*
**TV1**	TV1 + CA1 + LC2	10.00$^{\#}$ + 33.30$^{\#\#}$ + 55.30$^{\#\#}$	15.50 ± 3.31*	42.00 ± 4.24	74.00 ± 7.78
**LC2**			15.03 ± 1.55*	44.80 ± 3.18	71.80 ± 1.06*
**TV1**	TV1 + CA1 + LC3	10.00$^{\#}$ + 33.30$^{\#\#}$ + 5.53$^{\#\#}$	17.57 ± 3.91*	40.50 ± 2.83*	66.00 ± 2.83*
**TV1**	TV1 + CA1 + LC4	10.00$^{\#}$ + 33.30$^{\#\#}$ + 0.55$^{\#\#}$	18.83 ± 4.61*	35.00 ± 1.41*	51.50 ± 2.12*
**TV1**	TV1 + CA1 + LC5	10.00$^{\#}$ + 33.30$^{\#\#}$ + 0.05$^{\#\#}$	17.70 ± 2.97*	35.00 ± 4.24*	5.00 ± 3.54*
**TV1**	TV1 + CA2 + LC1	10.00$^{\#}$ + 3.33$^{\#\#}$ + 553.00$^{\#\#}$	0.80 ± 0.46*	38.50 ± 2.12	79.50 ± 2.12*
**TV1**	TV1 + CA2 + LC2	10.00$^{\#}$ + 3.33$^{\#\#}$ + 55.30$^{\#\#}$	12.30 ± 0.99*	39.50 ± 2.12	72.00 ± 3.54*
**LC2**			17.87 ± 2.91*	41.80 ± 1.77	77.00 ± 3.54
**TV1**	TV1 + CA2 + LC3	10.00$^{\#}$ + 3.33$^{\#\#}$ + 5.53$^{\#\#}$	14.65 ± 1.34*	40.00 ± 1.41	71.00 ± 1.41*
**TV1**	TV1 + CA2 + LC4	10.00$^{\#}$ + 3.33$^{\#\#}$ + 0.55$^{\#\#}$	18.85 ± 3.61	35.00 ± 1.41*	63.50 ± 2.12*

**TV1**	TV1 + CA2 + LC5	$10.00^{\#} + 3.33^{\#\#} + 0.05^{\#\#}$	19.60 ± 3.11	35.50 ± 2.12*	0.00 ± 0.00*
**TV1**	TV1 + CA3 + LC1	$10.00^{\#} + 0.33^{\#\#} + 553.00^{\#\#}$	0.00 ± 0.00*	41.00 ± 8.13	87.30 ± 10.30
**CA3**			0.03 ± 0.06*	37.00 ± 0.71*	81.50 ± 0.71
**TV1**	**TV1 + CA3 + LC2**	$10.00^{\#} + 0.33^{\#\#} + 55.30^{\#\#}$	**26.30 ± 1.34**	**28.00 ± 6.36**	**80.00 ± 2.83**
**LC2**			**22.15 ± 2.15**	**37.00 ± 3.18**	**80.00 ± 0.00**
**CA3**			**24.17 ± 2.49**	**35.00 ± 1.41***	**78.00 ± 2.12**
**TV1**	TV1 + CA3 + LC3	$10.00^{\#} + 0.33^{\#\#} + 5.53^{\#\#}$	26.55 ± 1.34	42.00 ± 1.41*	69.50 ± 2.12*
**CA3**			29.47 ± 4.94	37.00 ± 1.41*	61.00 ± 1.41
**TV1**	TV1 + CA3 + LC4	$10.00^{\#} + 0.33^{\#\#} + 0.55^{\#\#}$	27.00 ± 3.25	40.50 ± 2.12	63.00 ± 4.24
**CA3**			31.13 ± 4.98	34.80 ± 2.47	61.00 ± 1.41
**TV1**	TV1 + CA3 + LC5	$10.00^{\#} + 0.33^{\#\#} + 0.05^{\#\#}$	29.97 ± 2.78	36.80 ± 1.06	0.00 ± 0.00*
**CA3**			32.03 ± 4.79	32.00 ± 1.41*	0.00 ± 0.00*
**TV1**	TV1 + CA4 + LC1	$10.00^{\#} + 0.03^{\#\#} + 553.00^{\#\#}$	0.00 ± 0.00*	37.00 ± 2.83	101.00 ± 1.41
**TV1**	TV1 + CA4 + LC2	$10.00^{\#} + 0.03^{\#\#} + 55.30^{\#\#}$	11.00 ± 0.71*	32.50 ± 0.70	68.80 ± 3.18*
**LC2**			5.17 ± 2.25*	30.80 ± 3.18	80.50 ± 0.71
**TV1**	TV1 + CA4 + LC3	$10.00^{\#} + 0.03^{\#\#} + 5.53^{\#\#}$	20.05 ± 1.77*	32.30 ± 5.30	56.50 ± 0.71
**TV1**	TV1 + CA4 + LC4	$10.00^{\#} + 0.03^{\#\#} + 0.55^{\#\#}$	25.60 ± 1.13	32.00 ± 6.36	52.50 ± 0.71*
**TV1**	TV1 + CA4 + LC5	$10.00^{\#} + 0.03^{\#\#} + 0.05^{\#\#}$	31.05 ± 0.55	31.50 ± 5.66	2.25 ± 0.35*
**TV1**	TV1 + CA5 + LC1	$10.00^{\#} + 0.003^{\#\#} + 553.00^{\#\#}$	0.00 ± 0.00*	31.00 ± 2.83	103.00 ± 0.71
**TV1**	TV1 + CA5 + LC2	$10.00^{\#} + 0.003^{\#\#} + 55.30^{\#\#}$	1.05 ± 0.21*	36.00 ± 7.10	71.50 ± 0.12*
**LC2**			0.73 ± 0.38*	23.00 ± 4.24	82.50 ± 1.77
**TV1**	TV1 + CA5 + LC3	$10.00^{\#} + 0.003^{\#\#} + 5.53^{\#\#}$	19.87 ± 1.56*	40.50 ± 2.12*	71.50 ± 0.12*
**TV1**	TV1 + CA5 + LC4	$10.00^{\#} + 0.003^{\#\#} + 0.55^{\#\#}$	26.43 ± 3.61	39.50 ± 0.71*	53.50 ± 4.95*
**TV1**	TV1 + CA5 + LC5	$10.00^{\#} + 0.003^{\#\#} + 0.05^{\#\#}$	31.57 ± 1.89	41.00 ± 2.83*	45.00 ± 7.10
**LC2**	TV2 + CA1 + LC2	$1.00^{\# +} + 33.3^{\#\#} + 55.30^{\#\#}$	2.97 ± 0.46	39.80 ± 2.47	80.00 ± 0.71
**LC2**	TV2 + CA2 + LC2	$1.00^{\# +} + 3.33^{\#\#} + 55.3^{\#\#}$	0.07 ± 0.06*	28.30 ± 0.35*	83.30 ± 0.35
**LC2**	TV2 + CA3 + LC2	$1.00^{\# +} + 0.33^{\#\#} + 55.30^{\#\#}$	0.00 ± 0.00*	13.30 ± 4.60*	79.80 ± 0.35
**CA3**			0.10 ± 0.14*	28.80 ± 0.35*	84.80 ± 3.89
**LC2**	TV2 + CA4 + LC2	$1.00^{\# +} + 0.03^{\#\#} + 55.30^{\#\#}$	0.00 ± 0.00*	0.25 ± 0.35*	80.50 ± 0.71
**LC2**	TV2 + CA5 + LC2	$1.00^{\# +} + 0.003^{\#\#}$ $55.30^{\#\#}$	0.00 ± 0.00*	0.25 ± 0.35*	79.80 ± 0.35
**CA3**	TV2 + CA3 + LC1	$1.00^{\# +} + 0.33^{\#\#}$ $553.00^{\#\#}$	0.00 ± 0.00*	11.30 ± 0.35*	85.30 ± 1.77
**CA3**	TV2 + CA3 + LC3	$1.00^{\# +} + 0.33^{\#\#}$ $5.53^{\#\#}$	2.55 ± 0.07*	36.00 ± 2.83	50.00 ± 3.54*
**CA3**	TV2 + CA3 + LC4	$1.00^{\# +} + 0.33^{\#\#}$ $0.55^{\#\#}$	4.05 ± 1.20	36.80 ± 4.60	46.80 ± 1.77

**CA3**	TV2 + CA3 + LC5	1.00$^{\# +}$ + 0.33$^{\#\#}$ 0.05$^{\#\#}$	4.70 ± 0.28	37.30 ± 4.60	43.30 ± 3.89

The selected combinations for subsequent assays, along with their means, are presented in bold text. The means of the monocultures of *T. vaginalis* (TV), *C. albicans* (CA), and *L. crispatus* (LC) at all densities were compared to the means of the co-cultures. A p-value of < 0.05 was considered statistically significant, with significant differences indicated by an asterisk (*).
